# Event-Based Fault-Tolerant Control for Wheel Driving Intelligent Vehicle System

**DOI:** 10.3390/s26196160

**Published:** 2026-09-29

**Authors:** Renyu Zhang, Renqiang Xie, Bin Guo

**Affiliations:** 1 Science and Technology Department, Xihua University, Chengdu 610039, China; zhangry@xhu.edu.cn; 2 Schlumberger Technology Services (Chengdu) Co., Ltd., Chengdu 610225, China; rxie4@slb.com; 3 College of Electrical Engineering, Sichuan University, Chengdu 610065, China

**Keywords:** intelligent vehicle, event-triggered control, actuator fault, tracking control, fault-tolerant control (FTC)

## Abstract

This study investigates a fault-tolerant control scheme for the autonomous intelligent vehicle, which is actuated by four independent motors. The main purpose is to realize the trajectory tracking control, to avoid collision accidents, and to save communication resources. First, by using the event-triggered sampled output, the state, fault efficiency index, and disturbance information are acquired through a novel observer. The convergence performance of the proposed observer is proved theoretically. Subsequently, to achieve the control purpose of this study, an event-based adaptive sliding mode fault-tolerant control scheme is proposed based on the estimated information. On the one hand, two adaptive parameters are included in the controller to reduce the work of parameter adjustment; on the other hand, two-channel event trigger generators are introduced to reduce the transmission load. Additionally, the estimated disturbance is also integrated into the controller scheme to enhance tracking precision and chattering attenuation. Finally, two cases are given to verify the fault tolerance and good tracking abilities of the proposed approach.

## 1. Introduction

Autonomous and self-driving intelligent vehicles have become important components of intelligent transportation systems. By integrating multi-sensor perception, motion planning, and closed-loop control, intelligent vehicles can achieve self-localization, environment recognition, and trajectory tracking, thereby improving driving safety, traffic efficiency, and passenger comfort [1,2,3]. In the vehicle trajectory planning system, from the control point of view, achieving the desired path can certainly save time and increase passenger comfort. The self-driving intelligent vehicle, which can achieve driving and braking independently, is a promising and flexible structure of the vehicle system. Reliability and safety are two fundamental issues for autonomous intelligent vehicle systems. In practical operation, actuator faults may occur in in-wheel motors due to overheating, mechanical degradation, inverter faults, excitation loss, or inaccurate measurements [4,5]. Such faults may reduce the effective control gain of the motor and directly deteriorate the tracking performance and operational safety of the vehicle. Moreover, external disturbances and model uncertainties, caused by varying road friction, parameter perturbations, load variations, and environmental changes, may further degrade the closed-loop performance. Therefore, it is necessary to develop a fault-tolerant control (FTC) method that can compensate for actuator faults and attenuate disturbances while maintaining satisfactory trajectory tracking performance.

FTC is an effective method to enhance the robustness and recovery system performance. When a fault occurs, the single-model vehicle self-performance recovery is critical. For the autonomous vehicle system, the motor actuator faults mainly come from excitation loss and inaccurate measurements, and the FTC aims to construct a reconfigurable scheme to compensate for the influences of the actuator fault and to maintain the desired tracking performance [6,7]. Some kinds of literature have been investigated on the vehicle system [8,9,10,11], and the FTC methods can be classified into passive and active approaches. For example, in [8,9], two active control efforts were investigated for electric vehicle systems based on the fault diagnosis (FD) technique; the torques were redistributed, and the optimal performances were guaranteed with the proposed methods. With the FD technique, the fault information can be obtained online, which in turn redistributes the control law in a timely manner. On the contrary, in the absence of fault information, abundant results have also been achieved. For instance, in [10], aiming at the vehicle system subjected to an actuator fault, a sliding mode control method was adopted to design the FTC method.

Note that the external disturbances cannot be eliminated completely and will also degrade the performance of the intelligent vehicle system [12,13]. Consequently, disturbance rejection is very important when constructing the control scheme. In practical driving scenarios, disturbances may be caused by communication fluctuations, variations in tire–road friction, parameter perturbations, load changes, and unmodeled dynamics. It should be noted that these disturbances do not necessarily enter the vehicle dynamics through the same channel as the control input. In other words, they may be mismatched disturbances, which cannot be directly suppressed by conventional matched-disturbance compensation or input-channel robust control techniques. Although disturbance rejection has been widely studied, the stability and tracking control of intelligent vehicle systems subject to mismatched disturbances have received relatively limited attention. This motivates the development of an observer-based fault-tolerant control scheme capable of estimating and compensating for lumped mismatched disturbances.

Sliding mode control (SMC) has been applied widely to improve the performance of industrial processes because of its robustness to unknown dynamics [13,14]. Recently, the emerging observer-based SMC has become an effective method to reduce jitter, and the outstanding feature of this approach is to utilize the estimated information to design the control algorithm [15,16,17]. For instance, in [15], Borja-Jaimes et al. designed a sliding mode observer to estimate the fault; the fault reconstruction performance was verified. However, the aforementioned results are based on the periodic sampling mechanism, i.e., the control law is updated in a fixed-period manner, which may lead to some unnecessary computations in the control process. Note the fact that the information transmissions will be reduced if only the necessary information is transmitted between the sensors and actuators [18,19]. Inspired by this, the event-triggered scheme has appeared in recent years, in which the sampled data is released only after a predesigned event condition is satisfied, and thus mitigates the waste of communication [20,21,22,23,24]. For example, in [20], a multi-area interconnected power system was investigated; the load frequency control integrated with the event-triggered SMC technique was constructed to ensure the stability of the system. However, in the above results [20,21], only one event trigger channel was contained. Moreover, there are no results reporting the event-based control for the intelligent vehicle system. For the autonomous intelligent vehicle system, on the one hand, each wheel is controlled independently, which implies that more actuators and sensors are embedded in the system. On the other hand, the surrounding environment changes frequently when the vehicle is operating, which means that more information will be transmitted. As the transmission channel and computation ability are limited, one of the control difficulties for the intelligent vehicle system is to find an effective way to reduce the communication load and to guarantee the trajectory following performance simultaneously. This also motivates the main results of this study.

This paper presents an even-triggered observer based adaptive SMC method for an autonomous intelligent vehicle system with vehicle faults and mismatched disturbance. The main contributions of the paper are as follows.

(1) An event-triggered composite observer is investigated for the intelligent vehicle system subject to actuator faults and lumped mismatched disturbances. Different from existing observer-based SMC/FTC methods that mainly rely on periodic sampling, the proposed observer reconstructs the vehicle states, actuator fault efficiency factor, and equivalent disturbance by using event-triggered sampled output. The convergence of the observer is theoretically guaranteed by explicitly considering the event-triggering error.
(2) An observer-based reconfigurable adaptive sliding mode FTC scheme is proposed by incorporating the estimated fault and disturbance information into the controller design. Different from the existing adaptive SMC/FTC law and event-triggered mechanisms. In this work, fault compensation, disturbance attenuation, adaptive sliding mode control, and chattering reduction are integrated into a unified framework. Moreover, two error-dependent adaptive parameters are introduced in the sliding mode manifold to improve convergence performance and reduce the parameter-tuning burden.
(3) A dual-channel event-triggered control architecture is established for the sensor and controller channels. The two triggering mechanisms play different roles: the former determines the update of the observer input, while the latter determines the update of the actuator control signal. Under the proposed event conditions, the communication load reduction and fault-tolerant tracking performance can be achieved simultaneously.

The remainder of this paper is as follows. Section 2 presents system modeling and problem formulation. Section 3 shows the observer-based event trigger SMC architecture. Section 4 gives two fault cases to show the validity of the method, and Section 5 concludes this study.

## 2. System Modeling and Problem Formulation

### 2.1. Dynamic Model of the Intelligent Autonomous Vehicle with Disturbance and Actuator Fault

The intelligent autonomous vehicle model can be shown in Figure 1. The single-track dynamic motion equations can be obtained as follows [9].

(1) $$\left\{\begin{array}{l} {\dot{V}}_{x}=V_{y}r-\frac{C_{a}}{m}V_{x}^{2}+\frac{F_{hl}^{x}+F_{hr}^{x}+\left(F_{ql}^{x}+F_{qr}^{x}\right)\cos\theta }{m}\\ \;-\frac{\left(F_{ql}^{y}+F_{qr}^{y}\right)\sin\theta }{m}+\zeta_{1}\left(t\right)\\ {\dot{V}}_{y}=-V_{x}r+\frac{-\left(F_{ql}^{y}+F_{qr}^{y}\right)\cos\theta -\left(F_{ql}^{x}+F_{qr}^{x}\right)\sin\theta }{m}\\ \;+\frac{F_{hl}^{y}+F_{hr}^{y}}{m}+\zeta_{2}\left(t\right)\\ \dot{r}=\frac{l_{s}}{I_{z}}\left(F_{ql}^{y}\sin\theta -F_{ql}^{x}\cos\theta +F_{qr}^{x}\cos\delta -F_{qr}^{y}\sin\theta \right)\\ \;+\frac{l_{s}}{I_{z}}\left(F_{hr}^{x}-F_{hl}^{x}\right)-\frac{l_{r}}{I_{z}}\left(F_{hl}^{y}+F_{hr}^{y}\right)\\ \;-\frac{l_{f}}{I_{z}}\left(F_{ql}^{y}+F_{qr}^{y}\right)\cos\theta -\frac{l_{f}}{I_{z}}\left(F_{ql}^{x}+F_{qr}^{x}\right)\sin\theta +\zeta_{3}\left(t\right) \end{array}\right.$$

where $V_{x}$ and $V_{y}$ represent the speeds for longitudinal and lateral direction, r is the yaw rate. ${F_{i}}^{x}$ and $F_{i}^{y}$ denote the tire forces of the longitudinal and lateral orientation, and $i=\left\{ql,qr,hl,hr\right\}$ represent the front left, front right, rear left, and rear right wheel, respectively. $l_{f}$ represents the distance between the front wheel and the center of the vehicle, $l_{r}$ stands for the distance between the rear wheel and the center of the vehicle, and $l_{s}$ is the one-half distance between the front two wheels. $I_{z}$ denotes the yaw moment of inertia, and m represents the vehicle mass. θ is the wheel steering angle. $C_{a}$ can represent the influence of aerodynamic drag. $\zeta_{h}\left(t\right)\left(h=1,2,3\right)$ are the external mismatched disturbances.

For the autonomous vehicle system, each wheel is an independent unit, and the unit can be formulated through a control gain $v_{i}=T_{i}/u_{i},\;i=\left\{ql,qr,hl,hr\right\}$, and $T_{i}$ denotes the output of the in-wheel torque, which is positive when driving and negative when braking, $u_{i}$ stands for the control input. In addition, for each wheel, the wheel dynamic system is modeled as $R_{f}{F_{i}}^{x}=T_{i}-I_{\omega }{\dot{w}}_{i}$, where $I_{\omega }$ is the wheel moment of inertia, $w_{i}$ denotes the angular speed, $R_{f}$ is the radius of each wheel.

In the intelligent vehicle system, a motor winding or an inverter phase fault may occur, which will reduce the performance of the wheel actuator; i.e., this kind of actuator fault can be considered as the performance degradation of the control gain, which is given as [9]

(2) $$v_{if}\left(t\right)=\left(1-\varsigma_{i}\right)v_{i}\left(t\right)+\varepsilon_{i}v_{si}\left(t\right)$$

where $\varsigma_{i}$ stands for the fault efficiency index that satisfies $\varsigma_{i}\in \left[\varsigma_{1},\;\varsigma_{2}\right]$, with $\varsigma_{1}\in \left[0,1\right)$ and $\varsigma_{2}\in \left(0,1\right)$. Assumed that ${\dot{\varsigma }}_{i}$ satisfies $\left\|{\dot{\varsigma }}_{i}\right\|\leq \varsigma_{a}$, where $\varsigma_{\alpha }>0$. $\varepsilon_{i}\in \left\{0,1\right\}$, implies that $\varepsilon_{i}=1$ represents the ith actuator is in a faulty condition and $\varepsilon_{i}=0$ indicates that the ith actuator is fault-free. $v_{si}\left(t\right)$ is the bias fault in the ith motor.

**Remark** **1.** *For the intelligent system, in this study, the control gain is used to represent the fault condition of the self-driving wheel, i.e., when the motor is fault-free, the condition *  $v_{ql}=v_{qr}=v_{hl}=v_{hr}=v_{m}$ *is satisfied, and* $v_{m}$ *presents the healthy condition. When a fault occurs,* $v_{if}\left(t\right)$ *is the current control gain.*

### 2.2. Control Problem Formulation

In the intelligent vehicle system, the tire force in the lateral direction can be presented as

(3) $$F_{q,h}^{y}=C_{q,h}\mu_{q,h}\left(V_{x},V_{y},r\right)$$

where $C_{q,h}$ denotes the front and rear tire cornering stiffness, $\mu_{q,h}\left(V_{x},V_{y},r\right)$ is calculated as follows

(4) $$\mu_{q}\left(V_{x},V_{y},r\right)=\theta -\frac{l_{f}r+V_{y}}{V_{x}},\mu_{h}\left(V_{x},V_{y},r\right)=\frac{l_{r}r-V_{y}}{V_{x}}$$

From (1)–(4), (1) can be rewritten as

(5) $$\dot{x}=G\left(x\right)+\frac{v_{m}}{R_{f}}B_{m}\left(\theta \right)\left(I-\lambda \left(\varsigma \right)\right)u+B_{y}\left(\theta \right)F_{y}+\omega \left(t\right)$$

where x denotes the states of the vehicle system, $G\left(x\right)$ is a nonlinear dynamic function, $B_{m}\left(\theta \right)$ and $B_{y}\left(\theta \right)$ stands for the control gain and the output dynamic gain, $\omega \left(t\right)$ presents the disturbances. The details of these parameters are shown as follows:

$$x={\left[V_{x}\;V_{y}\;r\right]}^{T},F_{y}={\left[F_{ql}^{y}\;F_{qr}^{y}\;F_{hl}^{y}\;F_{hr}^{y}\right]}^{T},\;G\left(x\right)={\left[G_{1}\left(x\right)\;G_{2}\left(x\right)\;G_{3}\left(x\right)\right]}^{T},$$

$$u={\left[u_{ql},u_{qr},u_{hl},u_{hr}\right]}^{T},\;\lambda \left(\varsigma \right)=diag\left\{\varsigma_{ql},\varsigma_{qr},\varsigma_{hl},\varsigma_{hr}\right\},$$

$$G_{1}\left(x\right)=V_{y}r-\frac{C_{a}}{m}V_{x}^{2}+\frac{2C_{f}l_{f}\sin\theta r+2C_{f}V_{y}\sin\theta }{mV_{x}},$$

$$G_{2}\left(x\right)=-V_{x}r+\frac{2\left(-C_{f}l_{f}\cos\theta +C_{r}l_{r}\right)r+2\left(-C_{r}+C_{f}\cos\theta \right)V_{y}}{mV_{x}},$$

$$G_{3}\left(x\right)=\frac{\left(2C_{f}l_{f}^{2}\cos\theta -2C_{r}{l_{r}}^{2}\right)r+\left(2C_{f}l_{f}\cos\theta +2C_{r}l_{r}\right)V_{y}}{I_{z}V_{x}},$$

$$B_{m}\left(\theta \right)=\left[\begin{array}{cccc} \frac{\cos\theta }{m} & \frac{\cos\theta }{m} & \frac{1}{m} & \frac{1}{m}\\ \frac{\sin\theta }{m} & \frac{\sin\theta }{m} & 0 & 0\\ {℘}_{1} & {℘}_{2} & -\frac{l_{s}}{I_{z}} & \frac{l_{s}}{I_{z}} \end{array}\right],\;B_{y}\left(\theta \right)=\left[\begin{array}{cccc} -\frac{\sin\theta }{m} & -\frac{\sin\delta }{m} & 0 & 0\\ \frac{\cos\theta }{m} & \frac{\cos\theta }{m} & \frac{1}{m} & \frac{1}{m}\\ \alpha_{1} & \alpha_{2} & -\frac{l_{r}}{I_{z}} & -\frac{l_{r}}{I_{z}} \end{array}\right],$$

$${℘}_{1}=\frac{-l_{s}\cos\theta }{I_{z}}-\frac{l_{f}\sin\theta }{I_{z}},\;{℘}_{2}=\frac{l_{s}\cos\theta }{I_{z}}-\frac{l_{f}\sin\theta }{I_{z}},\;\alpha_{1}=\frac{l_{s}\sin\theta }{I_{z}}-\frac{l_{f}\cos\theta }{I_{z}},$$

$$\alpha_{2}=\frac{-l_{s}\sin\theta }{I_{z}}-\frac{l_{f}\cos\theta }{I_{z}},\;\omega \left(t\right)=B_{m}\left(\theta \right)\left(-\frac{I_{\omega }}{R_{f}}\dot{w}+\frac{\varepsilon v_{s}\left(t\right)u}{R_{f}}\right)+{\left[\zeta_{1}\left(t\right),\zeta_{2}\left(t\right),\zeta_{3}\left(t\right)\right]}^{T},$$

$$v_{s}\left(t\right)=diag\left\{v_{si}\left(t\right)\right\},\;i=\left\{ql,qr,hl,hr\right\}.$$

**Remark** **2.** *From Equation (5), it can be checked that *  $G\left(x\right)$ *is a Lipschitz function, i.e.,* $G\left(c\right)-G\left(d\right)\leq K\left|c-d\right|$*, and* K *represents the Lipschitz constant.*

## 3. Proposed Observer-Based Control Method

This part aims to show the design procedure of the proposed FTC event-based adaptive fast sliding mode approach for an intelligent vehicle, which contains three steps. The dual-event-triggered structure is given first. In step two, a composite observer is designed by using the event-triggered output information. The estimated values are used to construct the sliding mode surface and the robust sliding mode FTC scheme in step three.

### 3.1. Event Detector Design

For the intelligent vehicle system, the sensor, controller, and actuator are connected by a CAN bus, i.e., the area network is used as a communication medium. When the vehicle operates on a smooth road, it is unnecessary to transmit the signal at every period time because the communication data has small fluctuations. In order to reduce the transmission load of the vehicle system, the dual-event-triggered scheme shown in Figure 2 is determined; two event-triggered mechanisms are considered in this scheme.

Denote the current measured output as $y\left(j\right)\left(j=0,1,\ldots \right)$, and the latest released output signal as $y\left(t_{k}\right)\left(k=0,\;1,\ldots ,t_{0}=0\right)$, k denotes the number of the event triggering. Then, the sensor-controller channel trigger scheme is given by

(6) $${\left[y\left(t_{k}\right)-y\left(t\right)\right]}^{T}\varphi \left[y\left(t_{k}\right)-y\left(t\right)\right]\leq \Lambda y{\left(t_{k}\right)}^{T}\varphi y\left(t_{k}\right)+\eta_{s}$$

where φ is a weighting matrix, $\Lambda \in \left(0,1\right)$ is a given parameter. According to condition (6), $\eta_{s}$ is a positive scalar. The next release time can be determined by

(7) $$t_{k+1}=t_{k}+\underset{l\;>\;0}{\min}\left\{l\left|{e_{y}}^{T}\varphi e_{y}>\Lambda y{\left(t_{k}\right)}^{T}\varphi y\left(t_{k}\right)+\eta_{s}\right.\right\}$$

where $e_{y}=y\left(t_{k}\right)-y\left(t\right)$.

Similarly, denote the current output of the controller and the latest released control as $u\left(t\right)$ and $u\left(t_{m}\right)\left(m=0,1\ldots \right)$, $t_{m}$ is the triggered time constant. The details of the controller-to-actuator channel scheme are described in (37)–(41).

**Remark** **3.** *In the event-triggered scheme of the intelligent vehicle system, the current and the latest released signals are stored in a register, and the comparators will calculate to judge whether the condition (6) is satisfied. When a motor fault occurs, the current output of the system will deviate from the latest outputs; if the motor fault degree is severe, the output is also severely affected. Once the trigger condition is satisfied, the sampled measurement is transmitted. One can also see that if the threshold becomes smaller, then the trigger condition can be more easily satisfied and the data will be updated frequently enough. On the contrary, when the threshold gets larger, the data will be released in a lower frequency way. From this point of view, only parts of the sampled outputs need to be transmitted, and thus the transmission load is reduced.*

**Assumption** **1.** *For the intelligent vehicle system, the external disturbances mainly describe the load and communication fluctuations; these terms are bounded. With the safety protection device, such as ABS, in this paper, assume the external disturbance *  $\zeta_{h}\left(t\right)\left(h=1,2,3\right)$ *and its derivative signal are bounded, i.e., *$\left\|\zeta_{h}\left(t\right)\right\|\leq \zeta_{m}$*,* $\left\|{\dot{\zeta }}_{h}\left(t\right)\right\|\leq \zeta_{n}$*, where *$\zeta_{m}>0$ *and* $\zeta_{n}>0$.

**Remark** **4.** *Based on the above assumption 1 and Equation (5), it can be checked that *  $\omega \left(t\right)$ *is bounded. As both the acceleration of the wheel angular velocity and the motor input signal of the vehicle system are bounded. It is easy to deduce that *$\dot{\omega }\left(t\right)$ *is also bounded, i.e., there exist two constants* $d_{k}$ *and* $d_{c}$ *with* $\left\|\omega \left(t\right)\right\|\leq d_{k}$*, and* $\left\|\dot{\omega }\left(t\right)\right\|\leq d_{c}$.

### 3.2. Proposed Observer Design

By using the $y\left(t_{k}\right)$ information, the following composite observer is proposed

(8) $$\left\{\begin{array}{l} \dot{\hat{x}}\left(t\right)=G\left(\hat{x}\right)+\frac{v_{m}}{R_{f}}B_{m}\left(\theta \right)\lambda \left(I-\left(\hat{\varsigma }\right)\right)u+B_{y}\left(\theta \right)F_{y}\\ \;+\hat{\omega }\left(t\right)+L\left(y\left(t_{k}\right)-\hat{y}\left(t\right)\right)\\ \dot{\hat{\omega }}\left(t\right)=-H\hat{\omega }\left(t\right)+H_{1}\left(y\left(t_{k}\right)-\hat{y}\left(t\right)\right)+\delta_{s}\left(t\right) \end{array}\right.$$

where $\hat{x}\left(t\right)$, $\hat{\omega }\left(t\right)$ and $\hat{\varsigma }\left(t\right)$ are the estimations of $x\left(t\right)$, $\omega \left(t\right)$ and $\varsigma \left(t\right)$ respectively. L L, H and $H_{1}$ are three observer gains that satisfy the condition in (16) and (17). $\delta_{s}\left(t\right)$ is a compensator which is defined as:

(9) $$\delta_{s}\left(t\right)=\frac{Qe_{\omega }}{\left\|e_{\omega }Q\right\|}\left(d_{k}+d_{c}\right)+\frac{Qe_{\omega }}{{\left\|e_{\omega }Q\right\|}^{2}}\left(e_{y}^{T}e_{y}+\mu_{k}\right)$$

where $\mu_{\kappa }=2\sum_{j=1}^{4}\left(\pi_{j}+\varepsilon_{j}\right)\left(\varsigma_{2}-\varsigma_{1}\right)\left(\varsigma_{2}-\varsigma_{1}+\varsigma_{\alpha }\right)$, $\varsigma_{a}$ is as defined in (2), $\varepsilon_{j}$ and $\pi_{j}$ are the jth diagonal elements of $z^{-1}$ and $z_{m}^{-1}$, z and $z_{m}$ are two diagonal matrices needed to be chosen later, $e_{\omega }=\omega \left(t\right)-\hat{\omega }\left(t\right)$, Q is a positive symmetry defined matrix that satisfy the condition in (17).

In the observer (8), the estimation law of $\hat{\varsigma }$ is designed as follows:

(10) $$\dot{\hat{\varsigma }}=\left\{\begin{array}{l} l_{k}\;\\ 0\;\hat{\varsigma }=1,\;\mathrm{and}\;l_{k}>0\;\mathrm{or}\;\hat{\varsigma }=\varsigma_{1},\;\mathrm{and}\;l_{k}<0 \end{array}\right.$$

(11) $${\dot{\hat{\varsigma }}}_{m}=\left\{\begin{array}{l} l_{m}\;\\ 0\;{\hat{\varsigma }}_{m}=1,\;\mathrm{and}\;l_{m}>0\;\mathrm{or}\;{\hat{\varsigma }}_{m}=\varsigma_{1},\;\mathrm{and}\;l_{m}<0 \end{array}\right.$$

where $l_{k}=z\left(-{\left(\lambda (u(t))\right)}^{T}B_{c}^{T}Pe_{x}-\left(\hat{\varsigma }-{\hat{\varsigma }}_{m}\right)\right)$, $l_{m}=z_{m}\left(\hat{\varsigma }-{\hat{\varsigma }}_{m}\right)$, $B_{c}=B_{m}\left(\theta \right)v_{m}/R_{f}$, $e_{x}=x\left(t\right)-\hat{x}\left(t\right)$, here, $\varsigma_{m}$ can be regarded as the output of ς.

By defining that $\tilde{\varsigma }=\varsigma -\hat{\varsigma }$, ${\tilde{\varsigma }}_{m}=\varsigma -{\hat{\varsigma }}_{m}$, then the following error dynamic systems can be calculated:

(12) $$\begin{array}{l} {\dot{e}}_{x}=G\left(x\right)-G\left(\hat{x}\right)-B_{c}\lambda \left(\tilde{\varsigma }\right)u+e_{\omega }-L\left(y\left(t_{k}\right)-\hat{y}\left(t\right)\right)\\ \;=G\left(x\right)-G\left(\hat{x}\right)-B_{c}\lambda \left(\tilde{\varsigma }\right)u+e_{\omega }-LCe_{x}\left(t\right)+Le_{y}\left(t\right) \end{array}$$

(13) $${\dot{e}}_{\omega }=-He_{\omega }\left(t\right)-H_{1}Ce_{x}\left(t\right)+H_{1}e_{y}\left(t\right)+H\omega \left(t\right)+\dot{\omega }\left(t\right)-\delta_{s}\left(t\right)$$

(14) $$\dot{\tilde{\varsigma }}=z\left({\left(\lambda (u(t))\right)}^{T}B_{c}^{T}Pe_{x}+\left(\hat{\varsigma }-{\hat{\varsigma }}_{m}\right)\right)+\dot{\varsigma }$$

(15) $${\dot{\tilde{\varsigma }}}_{m}=-z_{m}\left(\hat{\varsigma }-{\hat{\varsigma }}_{m}\right)+\dot{\varsigma }$$

In what follows, the convergence performance of the estimation errors is analyzed.

**Theorem** **1.** *Based on assumptions 1, observer (8), and estimation laws in (10) and (11), if there exist two positive symmetric matrices*  P *and* Q *, and the following inequalities hold, then the estimation errors of* $e_{x}$ *,* $e_{\omega }$ *,* $\tilde{\varsigma }$ *, and* ${\tilde{\varsigma }}_{m}$ *will converge to zero.*

(16) $$\frac{1}{\eta_{1}}PP+\eta_{1}K^{2}+2I+PLL^{T}P-PLC-L^{T}C^{T}P\leq -\varphi_{a}I$$

(17) $$P^{T}P+QH_{1}CC^{T}{H_{1}}^{T}Q+QHH^{T}Q-QH-H^{T}Q\leq -\varphi_{b}I$$

where $\varphi_{a}>0$ and $\varphi_{b}>0$, $\eta_{1}$ is a positive scalar.

**Proof.**  Choose the Lyapunov candidate function as

(18) $$V\left(t\right)={e_{x}}^{T}Pe_{x}+{e_{\omega }}^{T}Qe_{\omega }+z^{-1}{\tilde{\varsigma }}^{T}\tilde{\varsigma }+{z_{m}}^{-1}{{\tilde{\varsigma }}_{m}}^{T}{\tilde{\varsigma }}_{m}$$

From (14)–(17), it can be deduced that

(19) $$\begin{array}{cc} \dot{V}\left(t\right) & =2{e_{x}}^{T}P{\dot{e}}_{x}+2{e_{\omega }}^{T}Q{\dot{e}}_{\omega }+2z^{-1}{\tilde{\varsigma }}^{T}\dot{\tilde{\varsigma }}+2{z_{m}}^{-1}{{\tilde{\varsigma }}_{m}}^{T}{\dot{\tilde{\varsigma }}}_{m}\\ & =2{e_{x}}^{T}P\left(G\left(x\right)-G\left(\hat{x}\right)-B_{c}\lambda \left(\tilde{\varsigma }\right)u+e_{\omega }-LCe_{x}\left(t\right)\right)\\ & +2{e_{x}}^{T}PLe_{y}\left(t\right)+2{e_{\omega }}^{T}Q\left(-He_{\omega }\left(t\right)-H_{1}Ce_{x}\left(t\right)\right.\\ & \left.+H_{1}e_{y}\left(t\right)+H\omega \left(t\right)+\dot{\omega }\left(t\right)-\delta_{s}\left(t\right)\right)\\ & +z^{-1}{\tilde{\varsigma }}^{T}\left(z\left({\left(\lambda (u(t))\right)}^{T}B_{c}^{T}Pe_{x}+\left(\hat{\varsigma }-{\hat{\varsigma }}_{m}\right)\right)+\dot{\varsigma }\right)\\ & +2{z_{m}}^{-1}{{\tilde{\varsigma }}_{m}}^{T}\left(-z_{m}\left(\hat{\varsigma }-{\hat{\varsigma }}_{m}\right)+\dot{\varsigma }\right) \end{array}$$

From Young’s inequality, one has

(20) $$2{e_{x}}^{T}P\left(G\left(x\right)-G\left(\hat{x}\right)\right)\leq {\eta_{1}}^{-1}{e_{x}}^{T}PPe_{x}+\eta_{1}K^{2}{e_{x}}^{T}e_{x}$$

(21) $$2{e_{x}}^{T}Pe_{\omega }+2{e_{x}}^{T}PLe_{y}\leq {e_{x}}^{T}\left(I+PLL^{T}P\right)e_{x}+{e_{\omega }}^{T}P^{T}Pe_{\omega }+{e_{y}}^{T}e_{y}$$

(22) $$\begin{array}{l} 2{e_{\omega }}^{T}Q\left(-H_{1}Ce_{x}\left(t\right)+H_{1}e_{y}\left(t\right)\right)\\ \leq {e_{\omega }}^{T}\left(QH_{1}CC^{T}{H_{1}}^{T}Q+QHH^{T}Q\right)e_{\omega }+{e_{x}}^{T}e_{x}+{e_{y}}^{T}e_{y} \end{array}$$

It can be checked that

(23) $$2{e_{x}}^{T}PB_{c}\lambda \left(\tilde{\varsigma }\right)u=2{e_{x}}^{T}PB_{c}\lambda \left(u\right)\bar{\varsigma }$$

where $\lambda \left(u\right)=diag\left(u_{ql},u_{qr},u_{hl},u_{hr}\right)$, $\bar{\varsigma }={\left[{\bar{\varsigma }}_{ql},{\bar{\varsigma }}_{qr},{\bar{\varsigma }}_{hl},{\bar{\varsigma }}_{hr}\right]}^{T}$, and ${\bar{\varsigma }}_{i}=\varsigma_{i}-{\hat{\varsigma }}_{i},i=\left\{ql,qr,hl,hr\right\}$.

With Equations (20)–(23), (19) can be reformulated as

(24) $$\begin{array}{cc} \dot{V}\left(t\right) & \leq {e_{x}}^{T}\left({\eta_{1}}^{-1}PP+\eta_{1}K^{2}+2I+PLL^{T}P-PLC-L^{T}C^{T}P\right)e_{x}\\ & +{e_{\omega }}^{T}\left(P^{T}P+QH_{1}CC^{T}{H_{1}}^{T}Q+QHH^{T}Q-QH-H^{T}Q\right)e_{\omega }\\ & +2{e_{y}}^{T}e_{y}+2{e_{\omega }}^{T}Q\left(H\omega \left(t\right)+\dot{\omega }\left(t\right)-\delta_{s}\left(t\right)\right)\\ & +2{\tilde{\varsigma }}^{T}\left(\hat{\varsigma }-{\hat{\varsigma }}_{m}\right)+2z^{-1}\dot{\varsigma }+2{{\tilde{\varsigma }}_{m}}^{T}\left(\hat{\varsigma }-{\hat{\varsigma }}_{m}\right)+2{z_{m}}^{-1}{{\tilde{\varsigma }}_{m}}^{T}\dot{\varsigma } \end{array}$$

Note the fact that

(25) $$\begin{array}{l} {\tilde{\varsigma }}^{T}\left(\hat{\varsigma }-{\hat{\varsigma }}_{m}\right)-{{\tilde{\varsigma }}_{m}}^{T}\left(\hat{\varsigma }-{\hat{\varsigma }}_{m}\right)\\ ={\tilde{\varsigma }}^{T}\left(\hat{\varsigma }+\varsigma -\varsigma -{\hat{\varsigma }}_{m}\right)-{{\tilde{\varsigma }}_{m}}^{T}\left(\hat{\varsigma }+\varsigma -\varsigma -{\hat{\varsigma }}_{m}\right)\\ =-\left({\left\|{\tilde{\varsigma }}_{m}\right\|}^{2}-2{\tilde{\varsigma }}^{T}{\tilde{\varsigma }}_{m}+{\left\|\tilde{\varsigma }\right\|}^{2}\right)\leq 0 \end{array}$$

From (2), one can obtain that

(26) $$\begin{array}{cc} 2z^{-1}{\tilde{\varsigma }}^{T}\dot{\varsigma } & =2z^{-1}\left({\tilde{\varsigma }}^{T}\dot{\varsigma }+{\tilde{\varsigma }}^{T}\tilde{\varsigma }-{\tilde{\varsigma }}^{T}\tilde{\varsigma }\right)\\ & \leq -2z^{-1}{\bar{\varsigma }}^{T}\bar{\varsigma }+2\sum_{j=1}^{4}\varepsilon_{j}\left(\varsigma_{2}-\varsigma_{1}\right)\left(\varsigma_{2}-\varsigma_{1}+\varsigma_{\alpha }\right) \end{array}$$

(27) $$\begin{array}{cc} 2{z_{m}}^{-1}{{\tilde{\varsigma }}_{m}}^{T}\dot{\varsigma } & =2{z_{m}}^{-1}\left({{\tilde{\varsigma }}_{m}}^{T}\dot{\varsigma }+{{\tilde{\varsigma }}_{m}}^{T}{\tilde{\varsigma }}_{m}-{{\tilde{\varsigma }}_{m}}^{T}{\tilde{\varsigma }}_{m}\right)\\ & =-2{z_{m}}^{-1}{{\tilde{\varsigma }}_{m}}^{T}{\tilde{\varsigma }}_{m}+2{z_{m}}^{-1}{{\tilde{\varsigma }}_{m}}^{T}\left({\tilde{\varsigma }}_{m}+\dot{\varsigma }\right)\\ & \leq -2{z_{m}}^{-1}{{\tilde{\varsigma }}_{m}}^{T}{\tilde{\varsigma }}_{m}+2\sum_{j=1}^{4}\pi_{j}\left(\varsigma_{2}-\varsigma_{1}\right)\left(\varsigma_{2}-\varsigma_{1}+\varsigma_{\alpha }\right) \end{array}$$

Then, (24) can be rewritten as

(28) $$\begin{array}{cc} \dot{V}\left(t\right) & \leq {e_{x}}^{T}\left({\eta_{1}}^{-1}PP+\eta_{1}K^{2}+2I+PLL^{T}P-PLC-L^{T}C^{T}P\right)e_{x}\\ & +{e_{\omega }}^{T}\left(P^{T}P+QH_{1}CC^{T}{H_{1}}^{T}Q+QHH^{T}Q-QHH^{T}Q\right)e_{\omega }\\ & +2{e_{y}}^{T}e_{y}+2{e_{\omega }}^{T}Q\left(H\omega \left(t\right)+\dot{\omega }\left(t\right)-\delta_{s}\left(t\right)\right)-2{z_{m}}^{-1}{{\tilde{\varsigma }}_{m}}^{T}{\tilde{\varsigma }}_{m}\\ & -2z^{-1}{\bar{\varsigma }}^{T}\bar{\varsigma }+2\sum_{j=1}^{4}\left(\varepsilon_{j}+\pi_{j}\right)\left(\varsigma_{2}-\varsigma_{1}\right)\left(\varsigma_{2}-\varsigma_{1}+\varsigma_{\alpha }\right) \end{array}$$

From (9), one can obtain that

(29) $$\begin{array}{l} 2{e_{\omega }}^{T}Q\left(H\omega \left(t\right)+\dot{\omega }\left(t\right)-\delta_{s}\left(t\right)\right)+2{e_{y}}^{T}e_{y}\\ +2\sum_{j=1}^{4}\left(\varepsilon_{j}+\pi_{j}\right)\left(\varsigma_{2}-\varsigma_{1}\right)\left(\varsigma_{2}-\varsigma_{1}+\varsigma_{\alpha }\right)\\ \leq -\frac{2{e_{\omega }}^{T}QQe_{w}}{\left\|e_{w}Q\right\|}\left(d_{k}+d_{c}\right)-\frac{2{e_{\omega }}^{T}QQe_{w}}{{\left\|e_{w}Q\right\|}^{2}}\left(e_{y}^{T}e_{y}+\mu_{\kappa }\right)\\ \leq 0 \end{array}$$

It is not hard to check that two matrices $\Theta_{m}$ and $\Theta_{n}$ can be found to satisfy that

(30) $$-2z^{-1}\leq -\Theta_{m}I,\;-2z_{m}^{-1}\leq -\Theta_{n}I$$

Then, from (16) and (17), one has

(31) $$\dot{V}\left(t\right)\leq -\varphi_{a}{\left\|e_{x}\right\|}^{2}-\varphi_{b}{\left\|e_{\omega }\right\|}^{2}-\Theta_{m}{\left\|\bar{\varsigma }\right\|}^{2}-\Theta_{n}{\left\|{\tilde{\varsigma }}_{m}\right\|}^{2}$$

Defining a new vector as $ƛ={\left[{e_{x}}^{T},{e_{\omega }}^{T},{\bar{\varsigma }}^{T},{{\tilde{\varsigma }}_{m}}^{T}\right]}^{T}$, one can check that $\dot{V}\left(t\right)\leq -{ℜ}_{a}{\left\|ƛ\right\|}^{2}$, where ${ℜ}_{a}=\min\left\{\varphi_{a},\varphi_{b},\min\left\{\Theta_{m}\right\},\min\left\{\Theta_{n}\right\}\right\}$. From the definition of $V\left(t\right)$, it is not hard to deduce that two scalars $\underline{\kappa },\bar{\kappa }$ exist to satisfy that $\underline{\kappa }{\left\|ƛ\right\|}^{2}\leq V\left(t\right)\leq \bar{\kappa }{\left\|ƛ\right\|}^{2}$, which implies that $\dot{V}\left(t\right)\leq -\frac{{ℜ}_{a}}{\bar{\kappa }}V\left(t\right)$. Thus, according to the Lyapunov theorem, it can be concluded that the zero convergence properties of the estimation errors $e_{x}$, $e_{\omega }$, $\tilde{\varsigma }$ and ${\tilde{\varsigma }}_{m}$ can be achieved. □

**Remark** **5.** *The adaptive laws in (10) and (11) are designed for online fault-efficiency reconstruction. The switching-like form acts as a projection mechanism, which keeps the estimated fault efficiency factor within its admissible physical range and avoids parameter drift. Under the assumptions and matrix inequalities in Theorem 1, the augmented estimation error vector, including the fault-efficiency estimation errors, is guaranteed to converge. From the viewpoint of strict parameter identifiability, if the actuator input is persistently zero or lacks sufficient variation, different fault-efficiency factors may generate indistinguishable input–output responses, and exact fault-index identification cannot be guaranteed. In such a weak-excitation case, the projection-type adaptive laws keep the estimated fault efficiency factor within its admissible physical interval. Therefore, the observer can still provide bounded estimation signals and compensation information for the closed-loop FTC design.*

### 3.3. Event-Based Fault-Tolerant Controller Design

This subsection aims to design an event-triggered robust sliding mode FTC scheme to keep an acceptable tracking performance of the intelligent vehicle system by using the estimated information.

Define the generated trajectory as $x_{r}\left(t\right)$ then the tracking error can be obtained as

(32) $$e_{r}=\hat{x}\left(t\right)-x_{r}\left(t\right)$$

where $x_{r}\left(t\right)$ represents the generated trajectories $\left(V_{xr},V_{yr},r_{r}\right)$.

In this paper, the sliding mode manifold is designed as follows

(33) $$\begin{array}{cc} s\left(t\right) & =e_{r}+\alpha_{1}\int \beta_{1}\left(e_{r}\right){\left|e_{r}\right|}^{\alpha_{1}-1}sign\left(e_{r}\right)+\beta_{k}\int e_{r}dt\\ & +\alpha_{2}\int \beta_{2}\left(e_{r}\right){\left|e_{r}\right|}^{\alpha_{2}-1}sign\left(e_{r}\right) \end{array}$$

where $1<\alpha_{1}=q/p<2<\alpha_{2}$ with p and q are two odd numbers, $\beta_{k}>0$, $\beta_{1}\left(e_{r}\right)$ and $\beta_{2}\left(e_{r}\right)$ are two adaptive parameters, which are given by

(34) $$\beta_{1}\left(e_{r}\right)=\lambda_{1}\exp\left(-\tau \left|e_{r}\right|\right)/\left(1+\lambda_{1}\exp\left(-\tau \left|e_{r}\right|\right)\right)$$

(35) $$\beta_{2}\left(e_{r}\right)=\lambda_{2}\exp\left(\tau \left|e_{r}\right|\right)/\left(1+\lambda_{2}\exp\left(\tau \left|e_{r}\right|\right)\right)$$

where τ>0, $\lambda_{1}>1$, and $0<\lambda_{2}<1$.

The proposed sliding-mode manifold is not intended to increase structural complexity, but to provide an error-dependent smooth weighting mechanism between the terminal sliding term and the fast terminal sliding term. In conventional fast terminal sliding mode control, an additional high-order nonlinear term is introduced to accelerate the response for large tracking errors, but the fixed gains of the low-order and high-order terms usually need careful tuning. If these gains are selected too large, the control input may become aggressive and chattering may be amplified; if they are too small, the convergence speed and robustness may deteriorate.

Different from the sliding mode controllers in [25,26,27], here, two new adaptive parameters in (34–35) are designed to reduce the parameter adjustment work. In other words, when the tracking error $\left|e_{r}\right|$ is large, one can check that $\beta_{1}\left(e_{r}\right)$ converges to 0 and $\beta_{2}\left(e_{r}\right)$ converges to 1, which implies that the last term mainly contributes and less reaching time is achieved. When the tracking error trends to the desired point, i.e., $\left|e_{r}\right|$ is small, accordingly, in this condition, $\beta_{1}\left(e_{r}\right)$ converges to $\lambda_{1}/\left(1+\lambda_{1}\right)$, and $\beta_{2}\left(e_{r}\right)$ converge to $\lambda_{2}/\left(1+\lambda_{2}\right)$, which indicates that the two adaptive terms mainly work; this enhances the robustness when the trajectory reaches the sliding mode manifold.

From (33), one can obtain that

(36) $$\begin{array}{cc} \dot{s}\left(t\right) & =\dot{\hat{x}}\left(t\right)-{\dot{x}}_{r}\left(t\right)+\alpha_{1}\beta_{1}\left(e_{r}\right){\left|e_{r}\right|}^{\alpha_{1}-1}sign\left(e_{r}\right)\\ & +\alpha_{2}\beta_{2}\left(e_{r}\right){\left|e_{r}\right|}^{\alpha_{2}-1}sign\left(e_{r}\right)+\beta_{k}e_{r}\\ & =G\left(\hat{x}\right)+B_{c}\lambda \left(I-\left(\hat{\varsigma }\right)\right)u\;+B_{y}\left(\theta \right)F_{y}+\hat{\omega }\left(t\right)\\ & +L\left(y\left(t_{k}\right)-\hat{y}\left(t\right)\right)-{\dot{x}}_{r}\left(t\right)+\alpha_{1}\beta_{1}\left(e_{r}\right){\left|e_{r}\right|}^{\alpha_{1}-1}sign\left(e_{r}\right)\\ & +\alpha_{2}\beta_{2}\left(e_{r}\right){\left|e_{r}\right|}^{\alpha_{2}-1}sign\left(e_{r}\right)+\beta_{k}e_{r} \end{array}$$

Then, the controller can be obtained as

(37) $$\begin{array}{cc} u\left(t\right) & =-\lambda {\left(\left(I-\hat{\varsigma }\right)\right)}^{-1}{B_{c}}^{†}\left[G\left(\hat{x}\right)+\right.B_{y}\left(\theta \right)F_{y}+\hat{\omega }\left(t\right)+\beta_{k}e_{r}\\ & +\alpha_{1}\beta_{1}\left(e_{r}\right){\left|e_{r}\left(t\right)\right|}^{\alpha_{1}-1}sign\left(e_{r}\right)+L\left(y\left(t_{k}\right)-\hat{y}\left(t\right)\right)\\ & \left.+\alpha_{2}\beta_{2}\left(e_{r}\right){\left|e_{r}\left(t\right)\right|}^{\alpha_{2}-1}sign\left(e_{r}\right)-{\dot{x}}_{r}\left(t\right)+\mu_{s}sign\left(s\right)\right] \end{array}$$

where $\mu_{s}$ is a positive scalar, ${B_{c}}^{†}={\left({B_{c}}^{T}B_{c}\right)}^{-1}{B_{c}}^{T}$.

In this study, the event-triggered sliding mode controller is designed as follows

(38) $$u\left(t_{m}\right)=u_{1}\left(t_{m}\right)+u_{2}\left(t_{m}\right)$$

(39) $$\begin{array}{cc} u_{1}\left(t_{m}\right) & =-\lambda {\left(\left(I-\hat{\varsigma }\left(t_{m}\right)\right)\right)}^{-1}{B_{c}}^{†}\left[G\left(\hat{x}\left(t_{m}\right)\right)+\right.B_{y}\left(\theta \right)F_{y}\\ & +\hat{\omega }\left(t_{m}\right)+\alpha_{1}\beta_{1}\left(e_{r}\right){\left|e_{r}\left(t_{m}\right)\right|}^{\alpha_{1}-1}sign\left(e_{r}\right)+\beta_{k}e_{r}\left(t_{m}\right)\\ & +\alpha_{2}\beta_{2}\left(e_{r}\right){\left|e_{r}\left(t_{m}\right)\right|}^{\alpha_{2}-1}sign\left(e_{r}\right)\left.-{\dot{x}}_{r}\left(t_{m}\right)\right] \end{array}$$

where $\hat{x}\left(t_{m}\right)$ and $\hat{\omega }\left(t_{m}\right)$ are the latest released signals from the controller, $t_{m}$ is the triggered time constant. The event-triggered condition in the controller-actuator channel is given by

(40) $$\left\|e_{1}\right\|=\left\|\bar{h}\left(\hat{x}\left(t_{m}\right)-\hat{x}\left(t\right)\right)\right\|\leq \exp\left(-\kappa_{1}t\right)+\mu_{1}$$

(41) $$\left\|e_{2}\right\|=\left\|\bar{h}\left(\hat{\omega }\left(t_{m}\right)-\hat{\omega }\left(t\right)\right)\right\|\leq \exp\left(-\kappa_{2}t\right)+\mu_{2}$$

where $\bar{h}=\lambda_{\min}\left(\Psi \right)$, Ψ is a parameter that needs to be designed later. $\mu_{1}$ and $\mu_{2}$ are two positive scalars, $\kappa_{1}>0$ and $\kappa_{2}>0$.

Similarly, the next release time can be determined by

(42) $$\begin{array}{l} t_{m+1}=t_{m}+\underset{l_{m}\;>\;0}{\min}\left\{l_{m}\left|\left\|e_{1}\right\|>\exp\left(-\kappa_{1}t\right)+\mu_{1}\;\right.\right.\\ \left.\;or\;\left\|e_{2}\right\|>\exp\left(-\kappa_{2}t\right)+\mu_{2}\right\} \end{array}$$

In the vehicle tracking control system, the event-triggered control approach contains two aspects. The first one is to calculate the errors in (40) and (41); the second one is to update the controller. Once the trigger conditions are violated, the data is released, and the controller (38) is updated. Otherwise, no updating behavior is necessary until the next event is triggered.

Define an auxiliary term as

(43) $$\begin{array}{cc} \chi_{s} & =\left\|G\left(\hat{x}\right)-\Psi G\left(\hat{x}\left(t_{m}\right)\right)\right\|+\left\|\hat{\omega }\left(t\right)-\Psi \hat{\omega }\left(t_{m}\right)\right\|\\ & +\left\|\alpha_{1}\beta_{1}\left(e_{r}\right)\beta_{1}\left(e_{r}\right){\left|e_{r}\right|}^{\alpha_{1}-1}sign\left(e_{r}\right)+\alpha_{2}\beta_{2}\left(e_{r}\right)\right.{\left|e_{r}\right|}^{\alpha_{2}-1}\\ & -\alpha_{1}\Psi \beta_{1}\left(e_{r}\left(t_{m}\right)\right){\left|e_{r}\left(t_{m}\right)\right|}^{\alpha_{1}-1}sign\left(e_{r}\left(t_{m}\right)\right)\\ & \left.-\alpha_{2}\Psi \beta_{2}\left(e_{r}\left(t_{m}\right)\right){\left|e_{r}\left(t_{m}\right)\right|}^{\alpha_{2}-1}sign\left(e_{r}\left(t_{m}\right)\right)\right\|\\ & +\left\|\left(I-\Psi \right)B_{y}\left(\delta \right)F_{y}\left(\delta \right)\right\|+\left\|\Psi {\dot{x}}_{r}\left(t_{m}\right)-{\dot{x}}_{r}\left(t\right)\right\|\\ & +\beta_{k}\left\|e_{r}-\Psi e_{r}\left(t_{m}\right)\right\|+\left\|L\left(\hat{y}\left(t_{k}\right)-\hat{y}\left(t\right)\right)\right\| \end{array}$$

where $\Psi =B_{c}\lambda \left(\left(I-\hat{\varsigma }\left(t\right)\right)\right)\lambda {\left(\left(I-\hat{\varsigma }\left(t_{m}\right)\right)\right)}^{-1}{B_{c}}^{†}$. From the definition of (43), one can observe that $\chi_{s}$ is bounded by designing the event trigger conditions in (40) and (41) properly.

In this paper, $u_{2}\left(t_{m}\right)$ is designed as

(44) $$u_{2}\left(t_{m}\right)=-\lambda {\left(\left(I-\hat{\varsigma }\left(t_{m}\right)\right)\right)}^{-1}{B_{c}}^{†}\left(\Psi^{-1}{\hat{\chi }}_{s}+\xi_{s}\right)\frac{s}{\left\|s^{T}\right\|}$$

where ${\dot{\hat{\chi }}}_{s}=\gamma \left(\left\|s^{T}\right\|+\delta_{k}\right)\left(s\left(t\right)\neq 0\right)$, and when $s\left(t\right)=0$, ${\hat{\chi }}_{s}$ is updated by ${\hat{\chi }}_{s}=\gamma_{s}\left\|\eta_{s}\right\|+\beta_{s}$, and $\eta_{s}$ can be obtained by $k_{\tau }{\dot{\eta }}_{s}+\eta_{s}=sign\left(s\left(t\right)\right)$, where $\xi_{s}$, γ, $\gamma_{s}$, $\beta_{s}$, $k_{\tau }$ and $\delta_{k}$ are positive scalars.

From [28], with the controller (44) and the sliding mode manifold, one can obtain that the estimated term is bound, i.e., ${\hat{\chi }}_{s}\left(t\right)\leq \bar{\chi },\forall t>0$, and $\bar{\chi }>0$.

**Theorem** **2.** *Based on the sliding mode manifold (33) and controller in (38), the tracking error will converge to zero.*

**Proof.**  Choose the Lyapunov candidate function as follows

(45) $$V_{1}\left(t\right)=\frac{1}{2}s{\left(t\right)}^{T}s\left(t\right)+\frac{1}{2\gamma }{\left({\hat{\chi }}_{s}-\bar{\chi }\right)}^{2}$$

From Equation (36), the derivative of (45) can be obtained as

(46) $$\begin{array}{cc} {\dot{V}}_{1}\left(t\right) & =s{\left(t\right)}^{T}\left[G\left(\hat{x}\right)\;+B_{c}\lambda \left(\left(I-\hat{\rho }\right)\right)u+B_{y}\left(\theta \right)F_{y}+\hat{\omega }\left(t\right)\right.\\ & +L\left(y\left(t_{k}\right)-\hat{y}\left(t\right)\right)-{\dot{x}}_{r}\left(t\right)+\alpha_{1}\beta_{1}\left(e_{r}\right){\left|e_{r}\right|}^{\alpha_{1}-1}sign\left(e_{r}\right)\\ & \left.+\alpha_{2}\beta_{2}\left(e_{r}\right){\left|e_{r}\right|}^{\alpha_{2}-1}sign\left(e_{r}\right)+e_{r}\right]+\frac{1}{\gamma }\left({\hat{\chi }}_{s}-\bar{\chi }\right){\dot{\hat{\chi }}}_{s} \end{array}$$

Substituting the control laws (39) and (44) into (46) yields

(47) $$\begin{array}{cc} {\dot{V}}_{1}\left(t\right) & \leq s{\left(t\right)}^{T}\left(\chi_{s}+B_{c}\lambda \left(\left(I-\hat{\rho }\right)\right)u_{2}\left(t_{m}\right)\right)+\gamma^{-1}\left({\hat{\chi }}_{s}-\bar{\chi }\right){\dot{\hat{\chi }}}_{s}\\ & =s{\left(t\right)}^{T}\chi_{s}+\left({\hat{\chi }}_{s}-\bar{\chi }\right)\left(\left\|s^{T}\right\|+\delta_{k}\right)\\ & -s{\left(t\right)}^{T}B_{c}\lambda \left(\left(I-\hat{\rho }\right)\right)\lambda {\left(\left(I-\hat{\rho }\left(t_{m}\right)\right)\right)}^{-1}{B_{c}}^{†}\left(\Psi^{-1}{\hat{\chi }}_{s}+\xi_{s}\right)\frac{s}{\left\|s^{T}\right\|}\\ & \leq \left\|s{\left(t\right)}^{T}\right\|\chi_{s}-\left\|s^{T}\right\|{\hat{\chi }}_{s}-\Psi \xi_{s}\left\|s^{T}\right\|+\left({\hat{\chi }}_{s}-\bar{\chi }\right)\left\|s^{T}\right\|+\left({\hat{\chi }}_{s}-\bar{\chi }\right)\delta_{k}\\ & \leq -\bar{h}\xi_{s}\left\|s^{T}\right\|-\delta_{k}\left|\bar{\chi }-{\hat{\chi }}_{s}\right|\\ & \leq -\theta_{s}\sqrt{V_{1}\left(t\right)} \end{array}$$

where $\theta_{s}=\min\left\{\sqrt{2}\bar{h}\xi_{s},\sqrt{2}\delta_{k}\;\right\}$.

By integrating (47) from 0 to $T_{m}$, the reaching time is obtained as $T_{m}\leq 2\sqrt{V_{1}\left(0\right)}/\theta_{s}$, where $T_{m}$ denotes the reaching time. Thus, the sliding motion is established; when the sliding motion occurs, it can be checked that ${\dot{e}}_{r}=-\alpha_{1}\beta_{1}\left(e_{r}\right){\left|e_{r}\right|}^{\alpha_{1}-1}sign\left(e_{r}\right)-\alpha_{2}\beta_{2}\left(e_{r}\right){\left|e_{r}\right|}^{\alpha_{2}-1}sign\left(e_{r}\right)-\beta_{k}e_{r}$, considering the ith component of the error, one has ${\dot{e}}_{r,i}=-\beta_{k}e_{r,i}-\alpha_{1}\beta_{1}(e_{r,i})|e_{r,i}{|}^{\alpha_{1}-1}\mathrm{sgn}(e_{r,i})-\alpha_{2}\beta_{2}(e_{r,i})|e_{r,i}{|}^{\alpha_{2}-1}\mathrm{sgn}(e_{r,i})$. From this expression, to obtain a conservative upper bound on convergence time, only one negative term that is sufficient to ensure finite-time convergence can be retained. Here, let $\;z_{i}=|e_{r,i}|$. Then one can easily check that ${\dot{z}}_{i}=\;\mathrm{sgn}\left(e_{r,i}\right){\dot{e}}_{r,i}\leq -\alpha_{1}{ℑ}_{lower}z_{i}^{\alpha_{1}-1}$, where ${ℑ}_{lower}>0$ stands for the lower bound of $\beta_{1}(e_{r,i})$. By integration, the settling time of $e_{r,i}$ after reaching the sliding manifold satisfies $T_{e,i}\leq \frac{|e_{r,i}(T_{s}){|}^{2-\alpha_{1}}}{\alpha_{1}{ℑ}_{lower}(2-\alpha_{1})}$. Therefore, the total finite-time convergence bound can be given by $T\leq T_{s}+\underset{i}{\max}\frac{|e_{r,i}(T_{s}){|}^{2-\alpha_{1}}}{\alpha_{1}{ℑ}_{lower}(2-\alpha_{1})}$. This completes the proof. □

**Remark** **6.** *From the control law (39), as* $\alpha_{1}>1$

*,* $\alpha_{2}>2$*, then the controller does not contain the infinity term, which means that the phenomenon of singularity can be avoided. Additionally, as the control law in (44) has the discontinuous term, one can implement the control law as (44) to reduce the chattering*

(48) $$u_{2}\left(t_{m}\right)=-\lambda {\left(\left(I-\hat{\varsigma }\left(t_{m}\right)\right)\right)}^{-1}{B_{c}}^{†}\left(\Psi^{-1}{\hat{\chi }}_{s}+\xi_{s}\right)\frac{s}{\left\|s^{T}\right\|+\rho_{k}}$$

where $\rho_{k}$ is small and $\rho_{k}>0$.

## 4. Simulation Results Analysis

The proposed scheme for the intelligent vehicle system is simulated with Simulink in this part; the vehicle parameters are given as m=1416 kg, $l_{s}=5.8695\;m$, $l_{f}=1.016\;m$, $l_{r}=1.562\;m$, $C_{a}=0.5$, $I_{z}=\begin{array}{l} 1523\;kg\cdot m^{2} \end{array}$, $R_{f}=0.311\;m$, $C_{f}=C_{r}=25796\;N/rad$. Two cases are considered in this subsection.

**Case** **1.** *In this case, the straight-line acceleration process is simulated, and the loss effectiveness of actuator fault and bias fault are considered, i.e.,* $\varepsilon_{i}=1$

*,* $0<\varsigma_{1}\leq \varsigma_{i}\leq \varsigma_{2}<1$ *. Assume the disturbances as*

$$\zeta_{1}\left(t\right)=\left\{\begin{array}{l} 5\cos(2t+6)(0\leq t<10)\\ 2\cos(2t-1)(10\leq t\leq 20) \end{array}\right.,\;\zeta_{2}\left(t\right)=\left\{\begin{array}{l} 0.5\sin(2t+3)\;(0\leq t<8)\\ 1\;(8\leq t\leq 20) \end{array}\right.,$$

$$\zeta_{3}\left(t\right)=\left\{\begin{array}{l} 0\;(0\leq t<6)\\ 1.5\sin(0.5t+5)(6\leq t\leq 20) \end{array}\right..$$

The bias faults are assumed as $v_{sql}\left(t\right)=\left\{\begin{array}{l} 0.1\sin(2t)(0\leq t<5)\\ 0.3\;(5\leq t\leq 20) \end{array}\right.$,

$$v_{sqr}\left(t\right)=\left\{\begin{array}{l} 0.02\cos(0.5t+1)\;(0\leq t<8)\\ 0.01\;(8\leq t\leq 20) \end{array}\right.,$$

$$v_{shl}\left(t\right)=\left\{\begin{array}{l} 0.01\;(0\leq t<10)\\ 0.02\;(10\leq t\leq 20) \end{array}\right.,\;v_{shr}\left(t\right)=\left\{\begin{array}{l} 0.03\;(0\leq t<15)\\ 0.1\;(15\leq t\leq 20) \end{array}\right..$$

Here, the fault index is given as $\varsigma \left(t\right)=\left\{\begin{array}{l} 0\;\left(0\leq t<8\right)\\ 0.57\;\left(8\leq t\leq 20\right) \end{array}\right.$.

The parameters in [11,25] are listed as $\delta_{c}\left(t\right)=\left\{\begin{array}{l} 0\;\left(0\leq t<4\right)\\ \sin\left(0.5\pi t\right)\;\left(4\leq t\leq 12\right)\\ 0\;\left(12\leq t\leq 20\right) \end{array}\right.$, $a_{xr}=0.6812$, $V_{y}=0.03\sin\left(0.5t\right)$.

The remaining parameters are chosen as follows, $\xi_{s}=0.1$, $\delta_{k}=0.2$, p=3, q=5, $\beta_{k}=10$, $\lambda_{1}=2.5$, $\lambda_{2}=0.5$, τ=2, $\kappa_{1}=20$, $\kappa_{1}=6$, $\mu_{1}=0.6$, $\mu_{2}=0.6$. By solving (16) and (17), one has $P=diag\left\{0.0898\;,0.0898\;,0.0898\;\right\}$, $L=diag\left\{\;7.6736,\;7.6736,7.6736\right\}$,

$$H=diag\left\{\;0.1797,\;0.1797,\;0.1797\right\},\;H_{1}=0.25.$$

The tracking performances are shown in Figure 3, Figure 4 and Figure 5.

In this case, the recently developed terminal sliding mode fault-tolerant control (TSMFTC) method in [26] and fast terminal sliding mode fault-tolerant control (FTSMFTC) method in [27] are also implemented for comparison on the intelligent vehicle system. The motion tracking results obtained by the three methods are shown in Figure 3, Figure 4 and Figure 5. As discussed above, the proposed method inherits the advantages of the methods in [26] and [27]. Moreover, the two introduced adaptive parameters further contribute to chattering attenuation and fault-tolerance enhancement. Consequently, the proposed method achieves improved tracking performance for the intelligent vehicle system.

Figure 6, Figure 7, Figure 8 and Figure 9 present the estimation results of the equivalent disturbances and the fault efficiency factor. It can be seen that the estimation errors remain relatively small during the motion process, which verifies the effectiveness of the designed observer and further supports the observer-based controller design. Figure 10 and Figure 11 show the release instants of the two event-triggered communication channels. At the initial stage of motion, more triggering events occur due to the larger control demand. As the tracking errors decrease, the triggering frequency is reduced; one can see that almost 89% and 63.6% of the communication data are saved in the sensor and actuator channels, indicating that the proposed event-triggered mechanism can effectively reduce computational and communication burdens.

Furthermore, the root mean square errors (RMSEs) of the three methods are summarized in Table 1. Compared with TSMFTC, the proposed method reduces the RMSEs of $e_{1}$, $e_{2}$, and $e_{3}$ from 0.0128, 0.0024, and 0.0014 to 0.0077, 0.0008, and 0.0006, corresponding to reductions of approximately 39.8%, 66.7%, and 57.1%, respectively. Compared with FTSMFTC, the proposed method decreases the RMSEs of $e_{1}$ and $e_{2}$ by approximately 27.4% and 38.5%, respectively, while the RMSE of $e_{3}$ remains at the same order of magnitude with only a slight increase from 0.0005 to 0.0006. These quantitative results further demonstrate that the proposed method provides improved overall tracking accuracy and stronger fault-tolerant performance. Figure 12 shows the motor output torque. From Figure 12, one can see that there was some fluctuation in the torque at the beginning, and it maintained good output performance afterwards with the proposed method.

**Case** **2.** *In this case, the straight-line acceleration and the uniform process are simulated, i.e., the high-speed condition is considered. The functions in* [11,25] *are assumed as*

$$\delta_{c}\left(t\right)=\left\{\begin{array}{l} 0\;\left(0\leq t<6\right)\\ \sin\left(0.5\pi t\right)\;\left(6\leq t\leq 16\right)\\ 0\;\left(16\leq t\leq 20\right) \end{array}\right.,\;V_{y}=0.02\sin\left(1.5t\right),\;\varsigma \left(t\right)=\left\{\begin{array}{l} 0\;\left(0\leq t<12\right)\\ 0.5+\sin\left(0.5\pi t\right)\left(12\leq t\leq 20\right) \end{array}\right.,$$

$$a_{xr}=\left\{\begin{array}{l} 2.7249\left(0\leq t<12.5\right)\\ 0\;\left(12.5\leq t<20\right) \end{array}\right..$$

The other parameters are the same as case 1.

In this case, the event-triggered integral sliding mode control (ET-ISMC) in [22] and event-based linear sliding mode control (ET-LSMC) method in [29] are considered for comparison. The tracking responses of the intelligent vehicle system under different controllers are shown in Figure 13, Figure 14 and Figure 15. It can be observed that all the compared methods can achieve trajectory tracking under the considered fault and disturbance conditions. However, compared with the controllers in [22,29], the proposed method exhibits faster response and smoother tracking behavior. In particular, the chattering phenomenon is effectively attenuated, which benefits from the adaptive design and the disturbance/fault compensation mechanism.

Figure 16, Figure 17 and Figure 18 show the disturbance estimation results obtained by the proposed observer and the sliding mode observer (SMO) method in [12]. As shown in these figures, the proposed observer achieves smaller estimation errors and better estimation accuracy, indicating its improved capability in reconstructing the unknown equivalent disturbances. Figure 19 presents the estimation result of the fault efficiency factor. The small estimation error further confirms the effectiveness of the proposed observer and supports the rationality of the observer-based fault-tolerant controller design. The release instants of the two event-triggered channels are shown in Figure 20 and Figure 21, respectively. It can be seen that when the number of transmissions is reduced one can see that almost 82.5% and 74.3% of the communication data are saved in the sensor and actuator channels, which demonstrates that the proposed event-triggered mechanism can decrease the communication burden while maintaining satisfactory control performance.

Furthermore, Figure 22 compares the tracking errors obtained by the proposed method and the other two methods, and the corresponding RMSE results are listed in Table 2. Compared with the method in [29], the proposed method reduces the RMSEs of $e_{1}$, $e_{2}$, and $e_{3}$ from 0.0255, 0.0030, and 0.0030 to 0.0219, 0.0007, and 0.0008, corresponding to reductions of approximately 14.1%, 76.7%, and 73.3%, respectively. Compared with the method in [22], the RMSEs of $e_{1}$, $e_{2}$, and $e_{3}$ are reduced by approximately 26.3%, 46.2%, and 42.9%, respectively. The simulation results further verify that the proposed method achieves better overall tracking accuracy, stronger disturbance rejection capability, and improved chattering suppression performance. Figure 23 shows the output motor torque. From Figure 23, one can see that there was also some fluctuation at the beginning, and it was redistributed with the proposed method.

## 5. Conclusions

In this study, an event-triggered robust sliding mode fault-tolerant control method is proposed for a class of intelligent autonomous vehicle systems with actuator faults and external disturbances. This problem is challenging due to the coexistence of nonlinear vehicle dynamics, unknown disturbances, motor efficiency degradation, communication constraints, and the chattering phenomenon of sliding mode control. To cope with these issues, a composite observer is designed to estimate the equivalent disturbances and the fault efficiency factor. Then, an adaptive sliding mode fault-tolerant controller is developed based on the estimated information, which improves fault compensation, disturbance attenuation, chattering reduction, and convergence performance. The reachability of the sliding surface is also theoretically guaranteed. Furthermore, two event-triggered schemes are introduced in the sensor-to-controller and controller-to-actuator channels to reduce unnecessary transmissions and communication burden. Two simulation cases are provided to validate the effectiveness of the proposed reconfigurable fault-tolerant control scheme. The comparative results demonstrate that the proposed method achieves better overall tracking accuracy, improved estimation performance, reduced chattering, and fewer communication transmissions than the existing methods.

It should be noted that the present simulation validation mainly focuses on verifying the core mechanism of the proposed event-triggered observer-based FTC scheme under actuator faults, lumped disturbances, and different speed conditions. For practical vehicle implementation, more complex driving scenarios and realistic constraints still need to be further considered, including varying road friction, curved trajectory tracking, lane-change maneuvers, sensor noise, actuator saturation, multi-wheel faults, parameter uncertainty, and real-time computational limitations. Future work will extend the proposed method from simulation to hardware-in-the-loop tests and real vehicle experiments, so as to further evaluate and improve the practical feasibility and engineering applicability of the proposed control strategy.

## Figures and Tables

**Figure 1:**
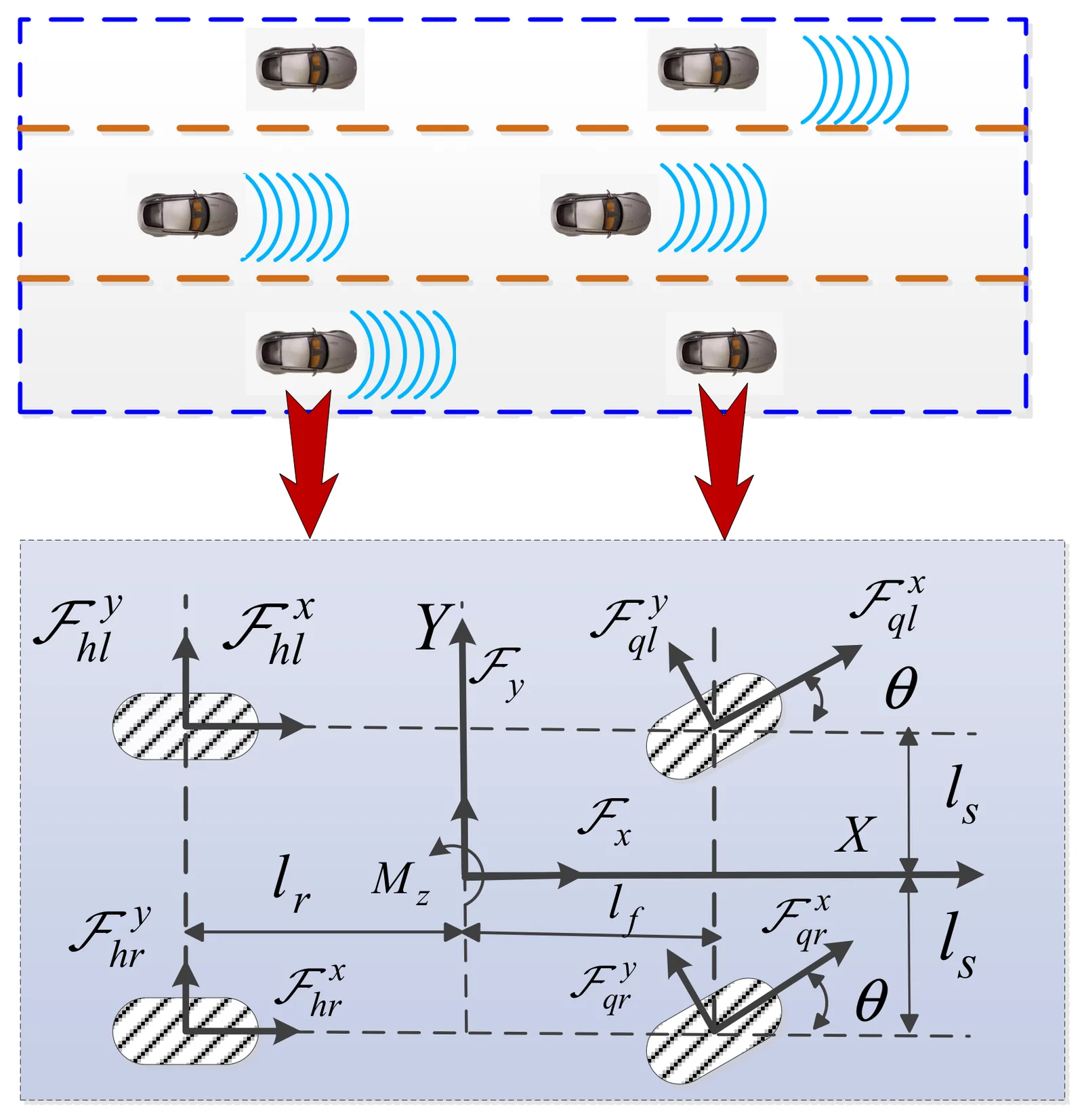
Schematic of the intelligent vehicle system.

**Figure 2:**
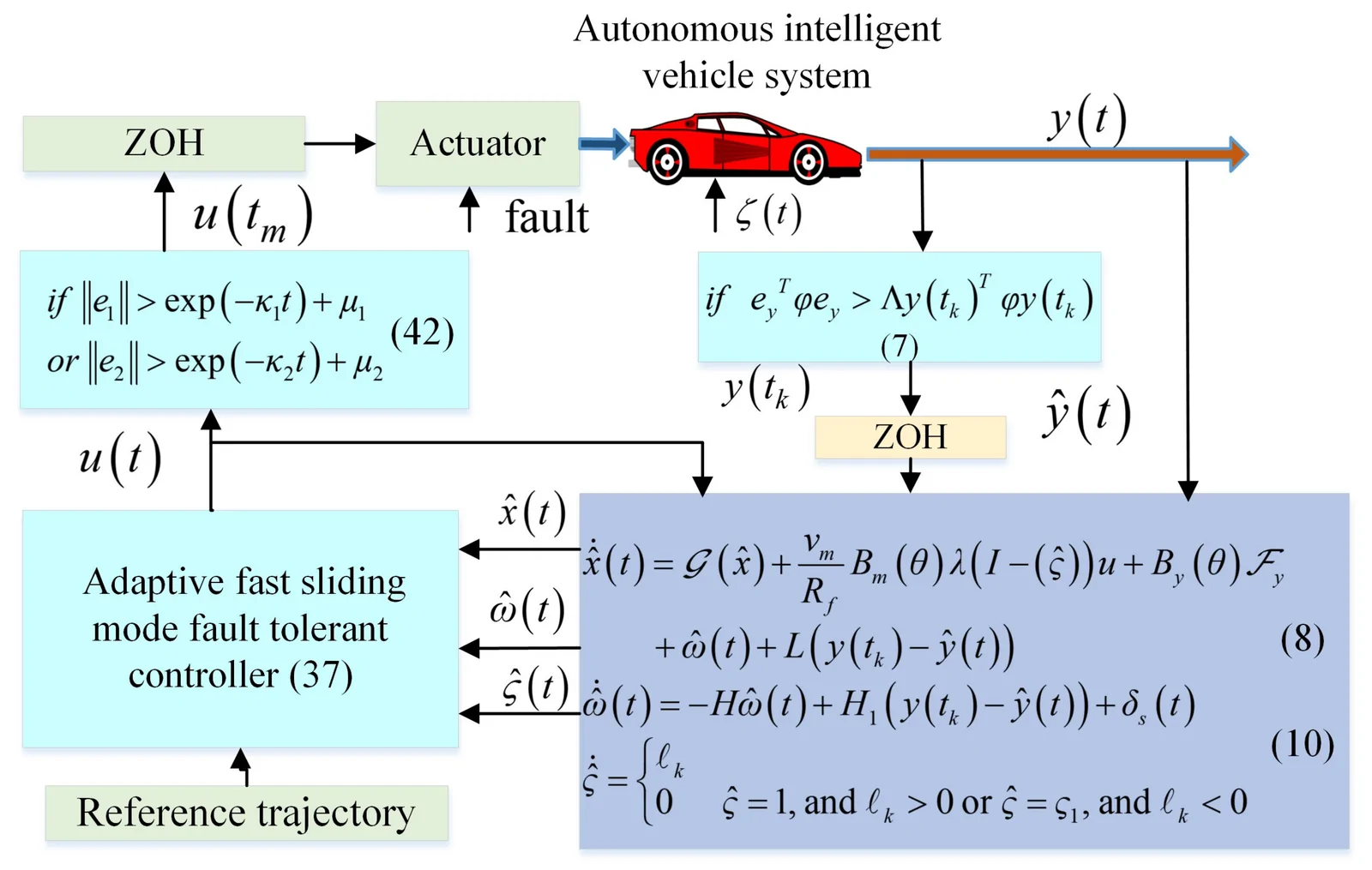
The control scheme of the study.

**Figure 3:**
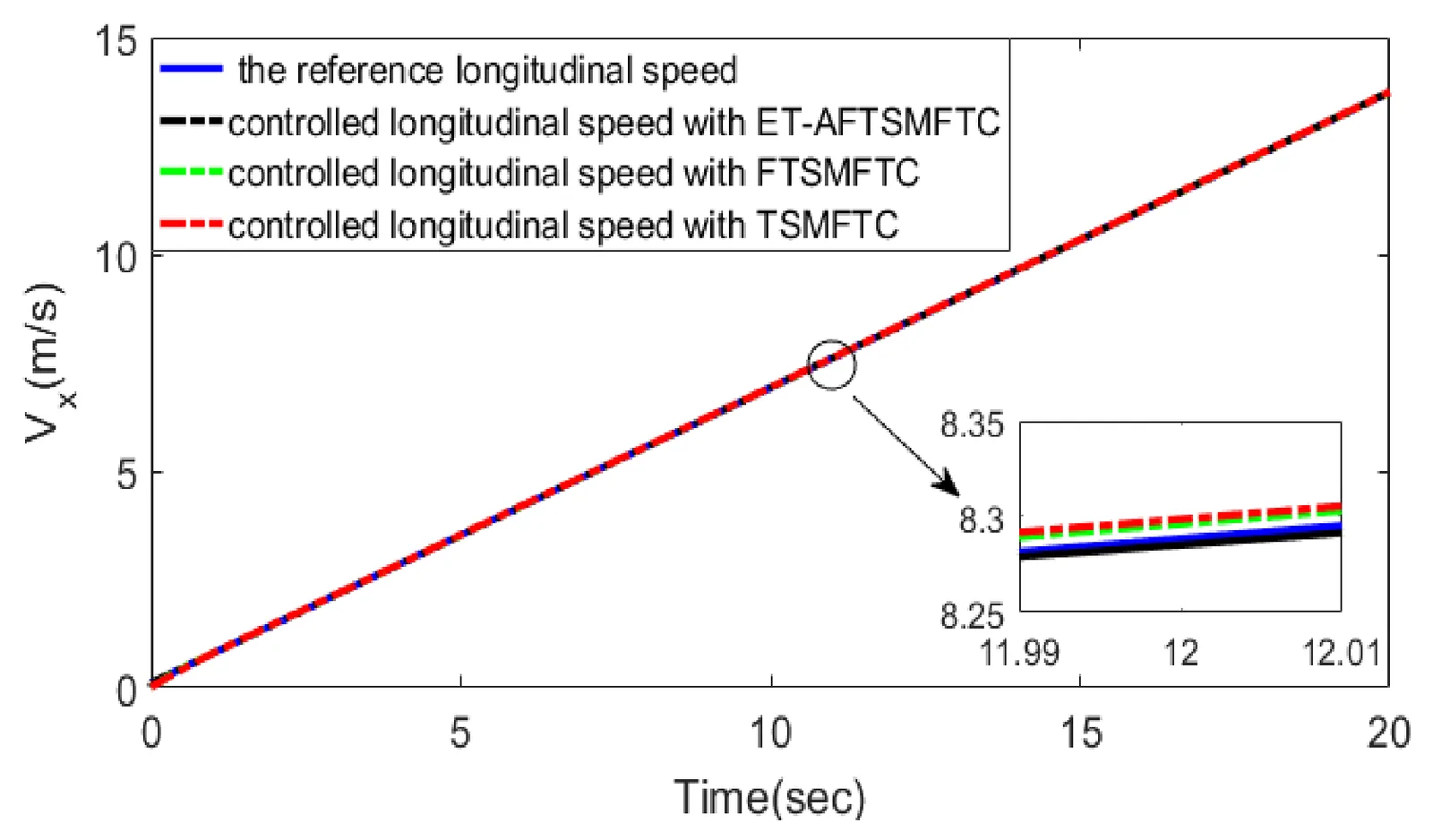
Longitudinal speed tracking results with three methods.

**Figure 4:**
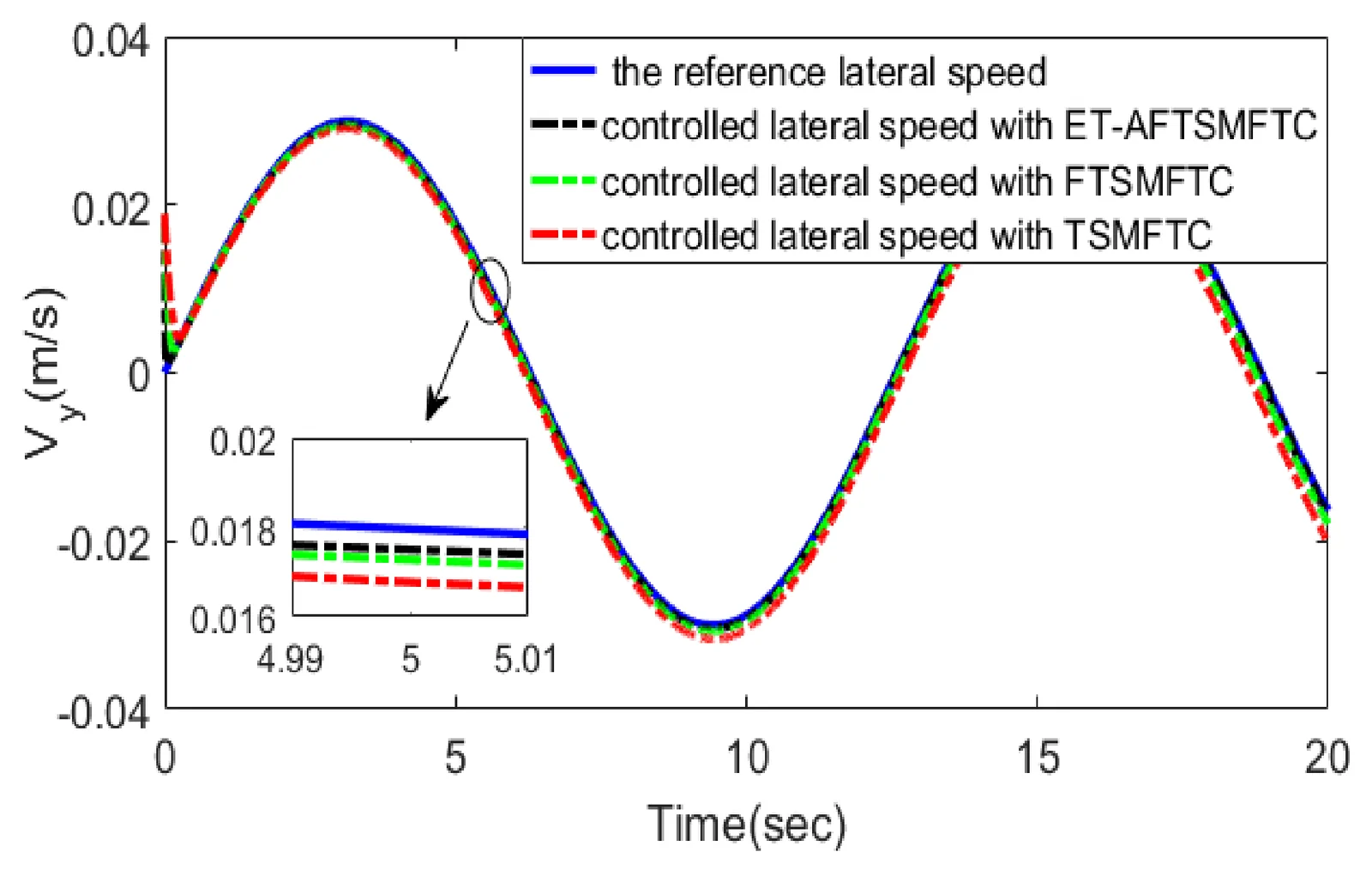
Lateral speed tracking results with different results.

**Figure 5:**
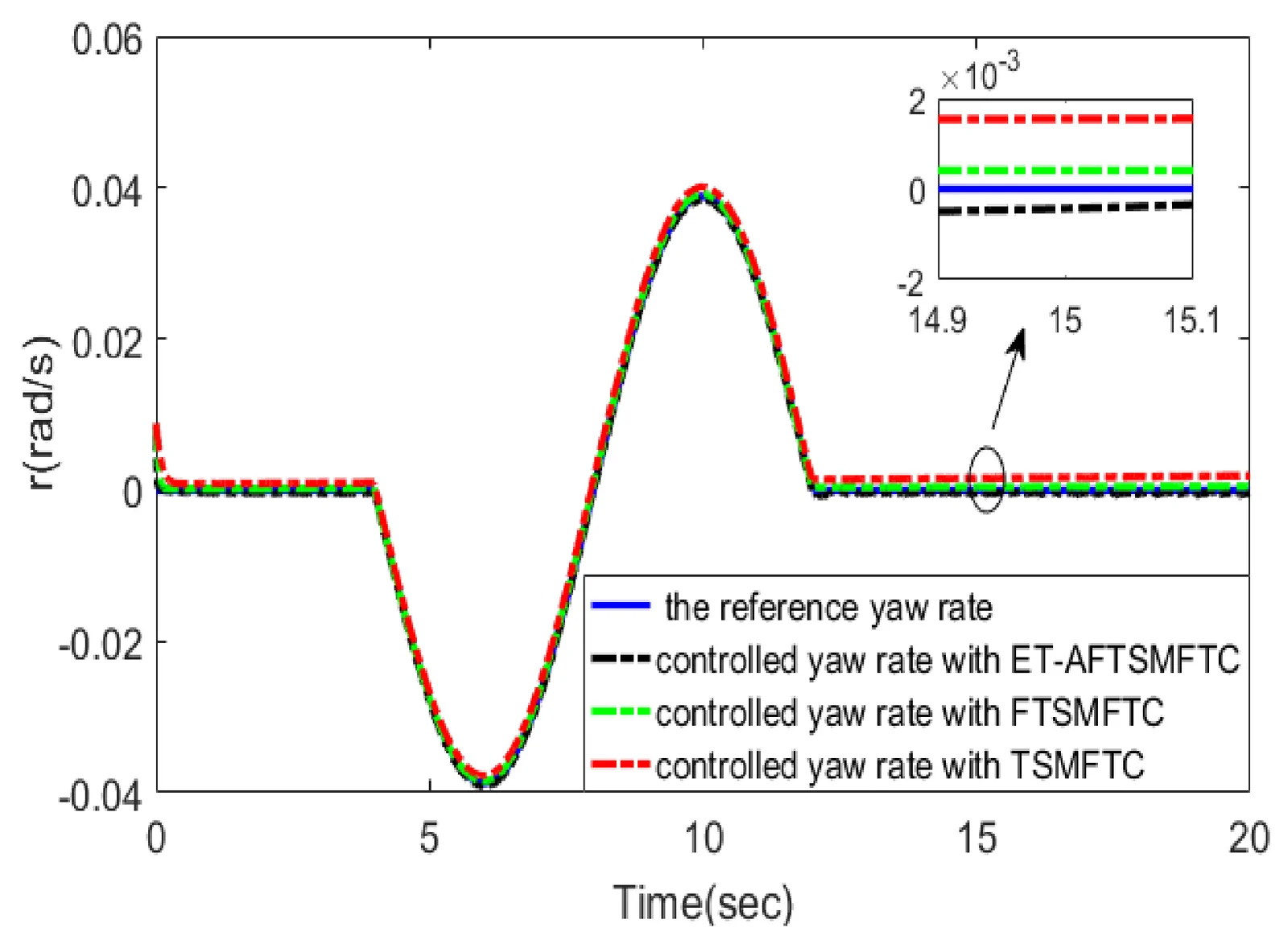
Yaw rate tracking results with different results.

**Figure 6:**
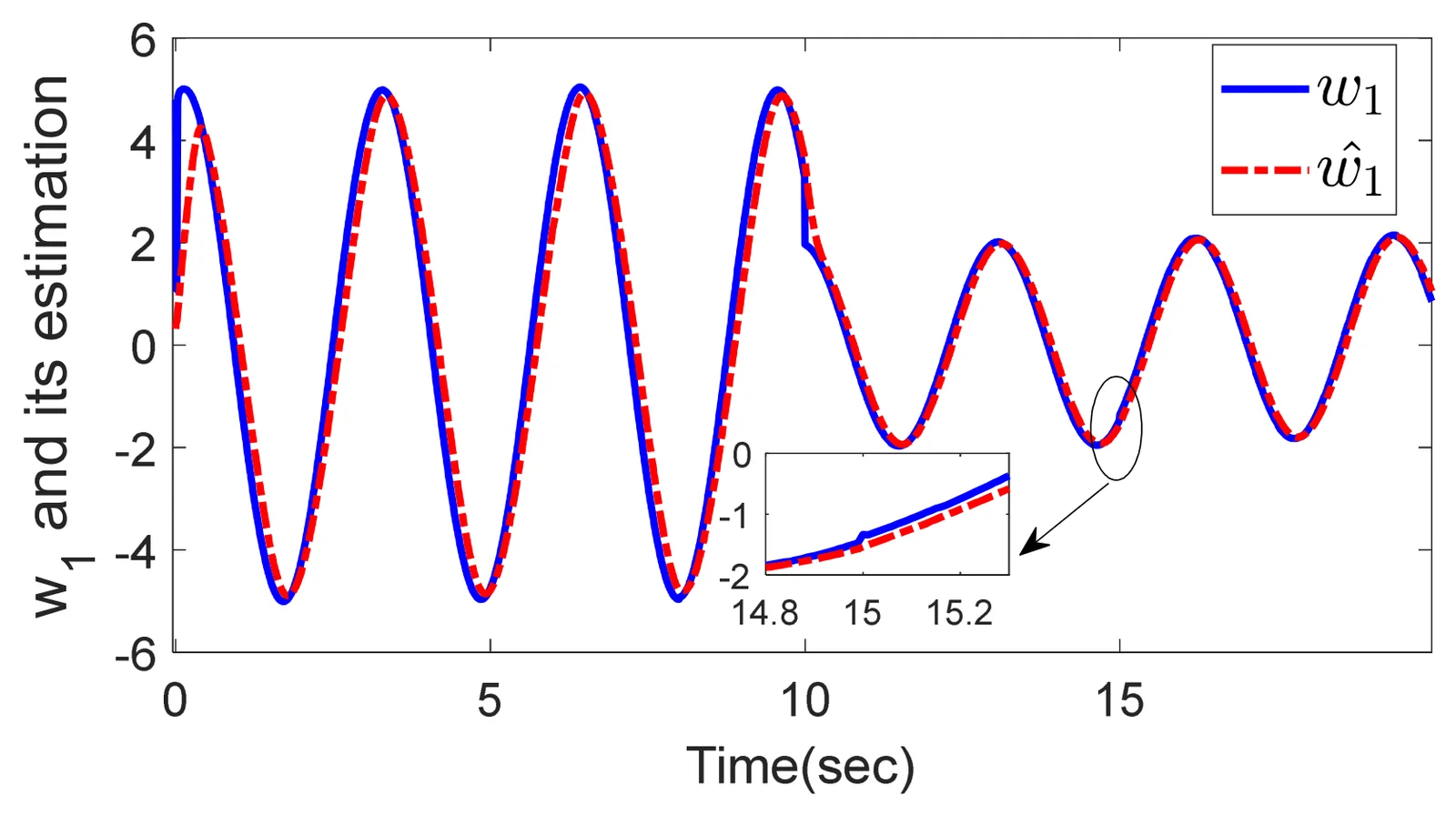
Equal disturbance $w_{1}$ and its estimation.

**Figure 7:**
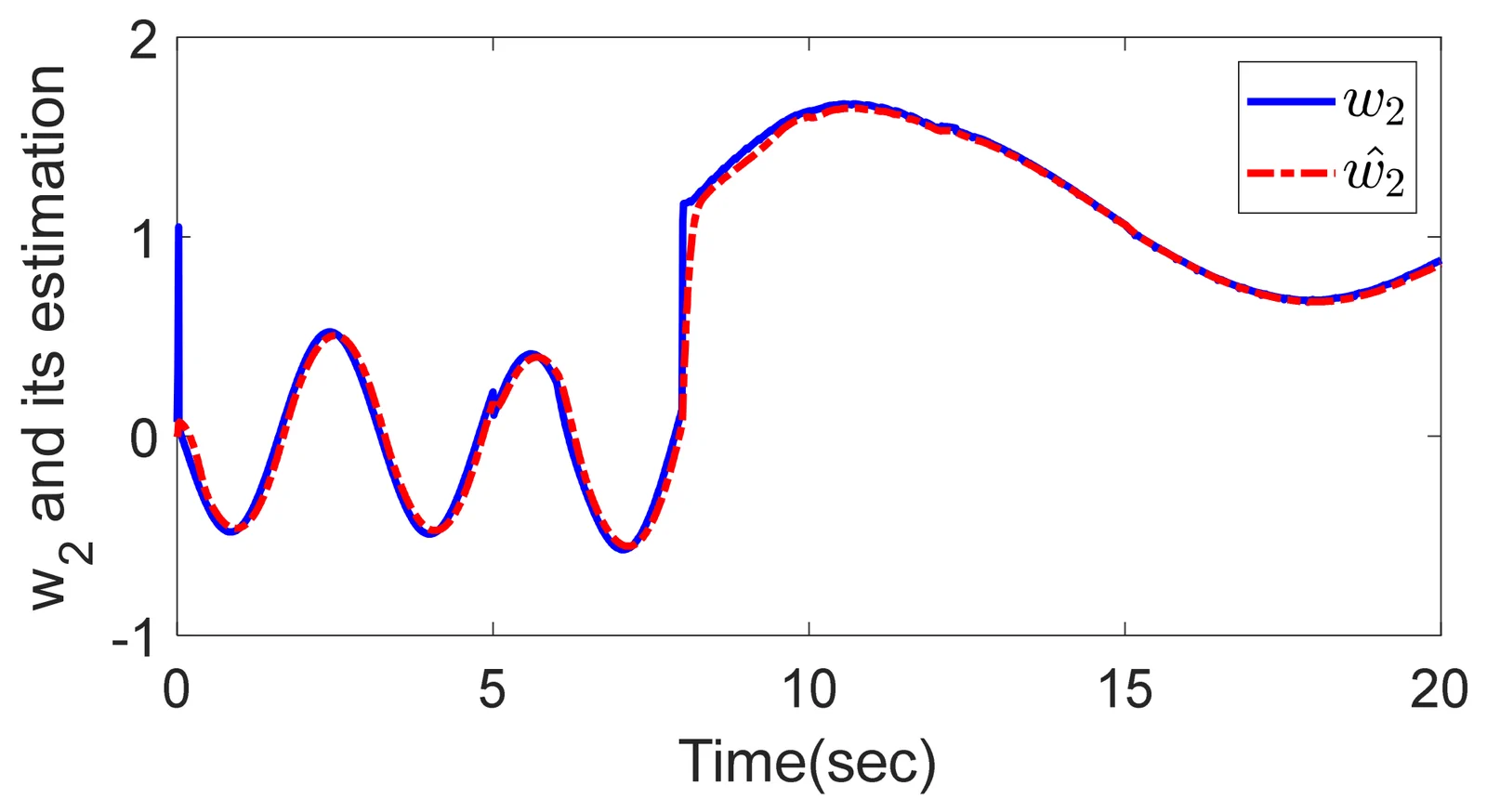
The disturbance $w_{2}$ and its estimation.

**Figure 8:**
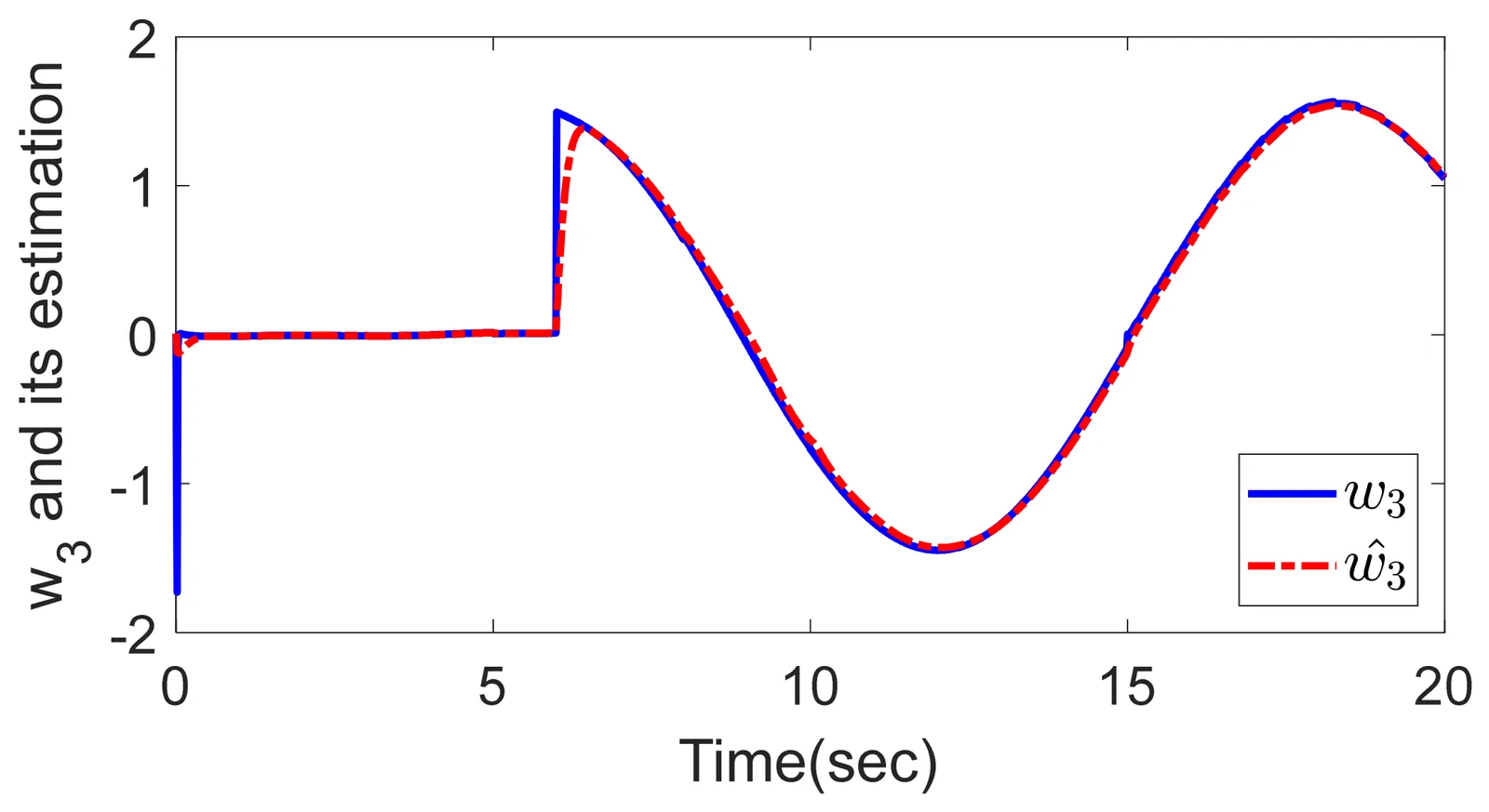
The disturbance $w_{3}$ and its estimation.

**Figure 9:**
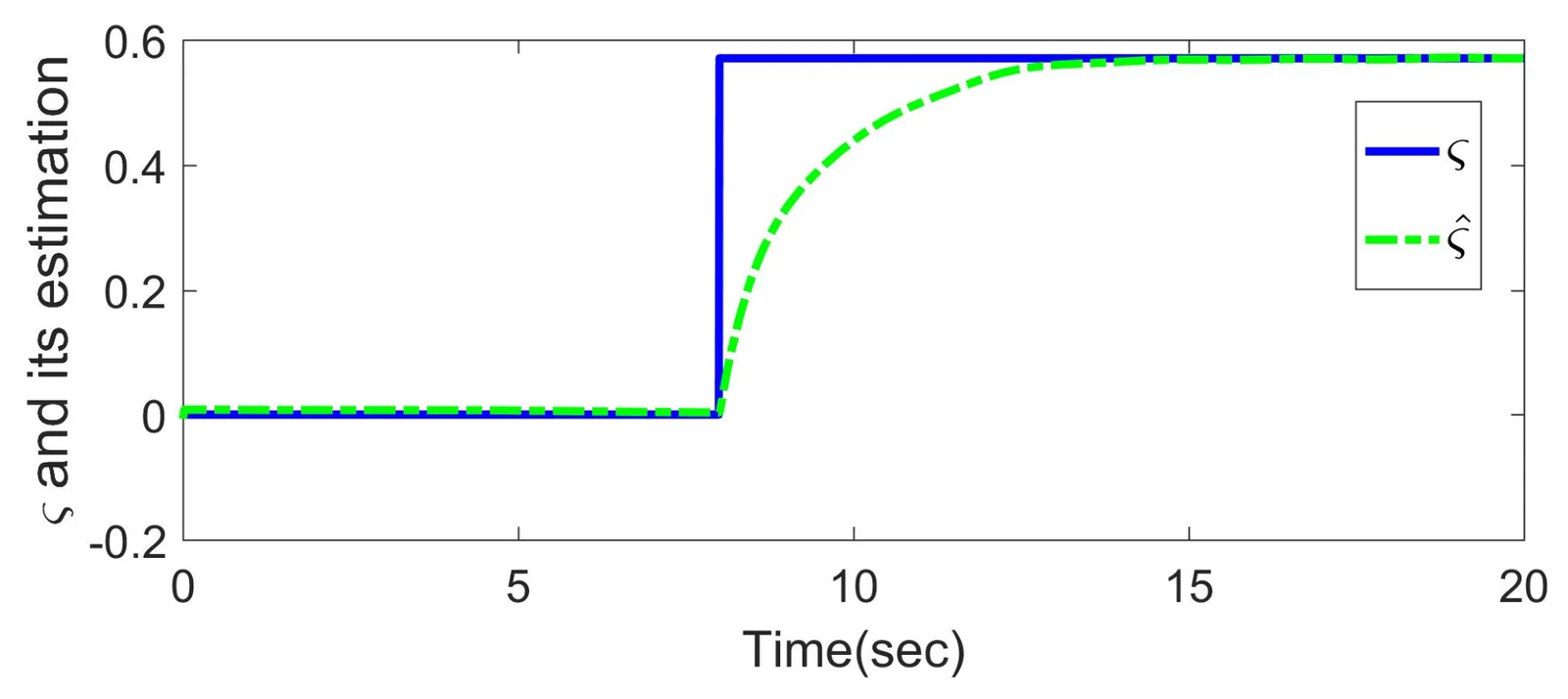
ς and its estimation result.

**Figure 10:**
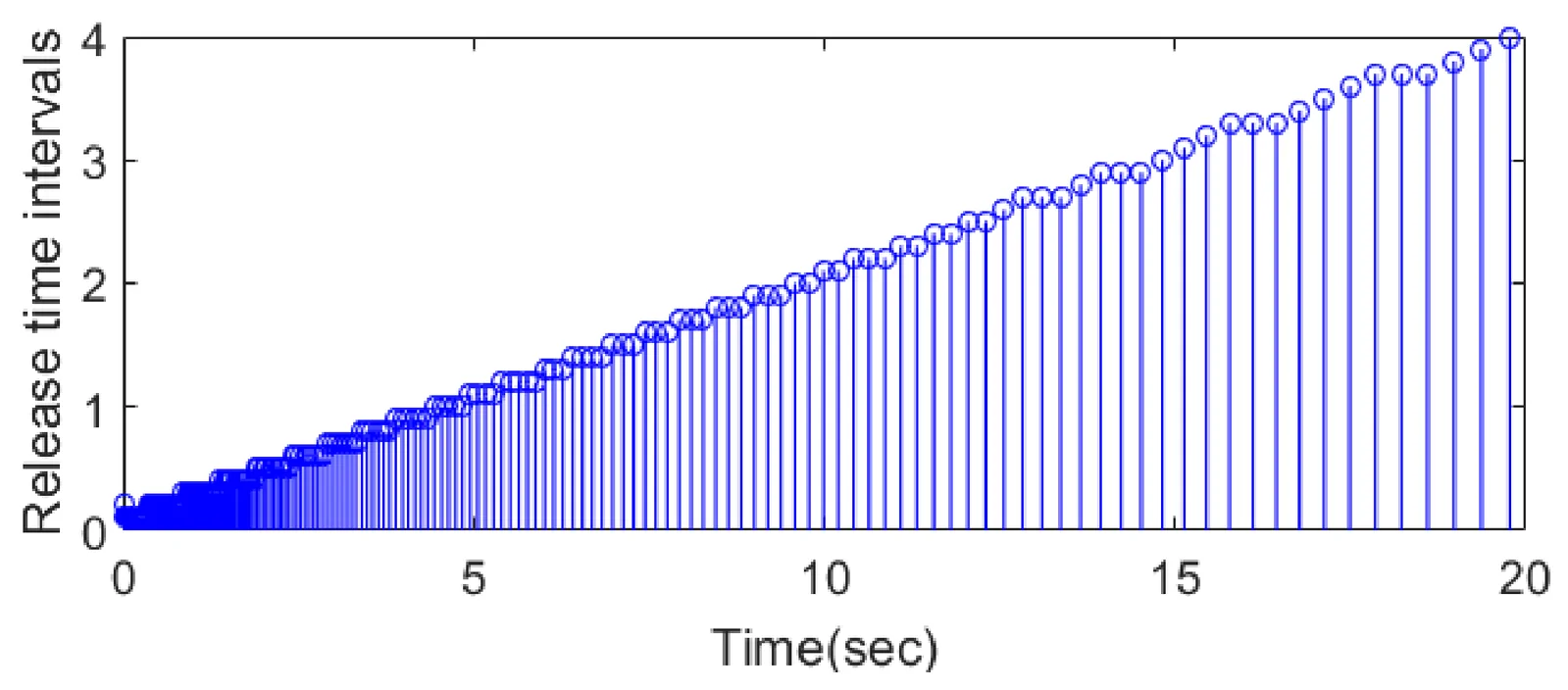
The release time intervals of output channel y.

**Figure 11:**
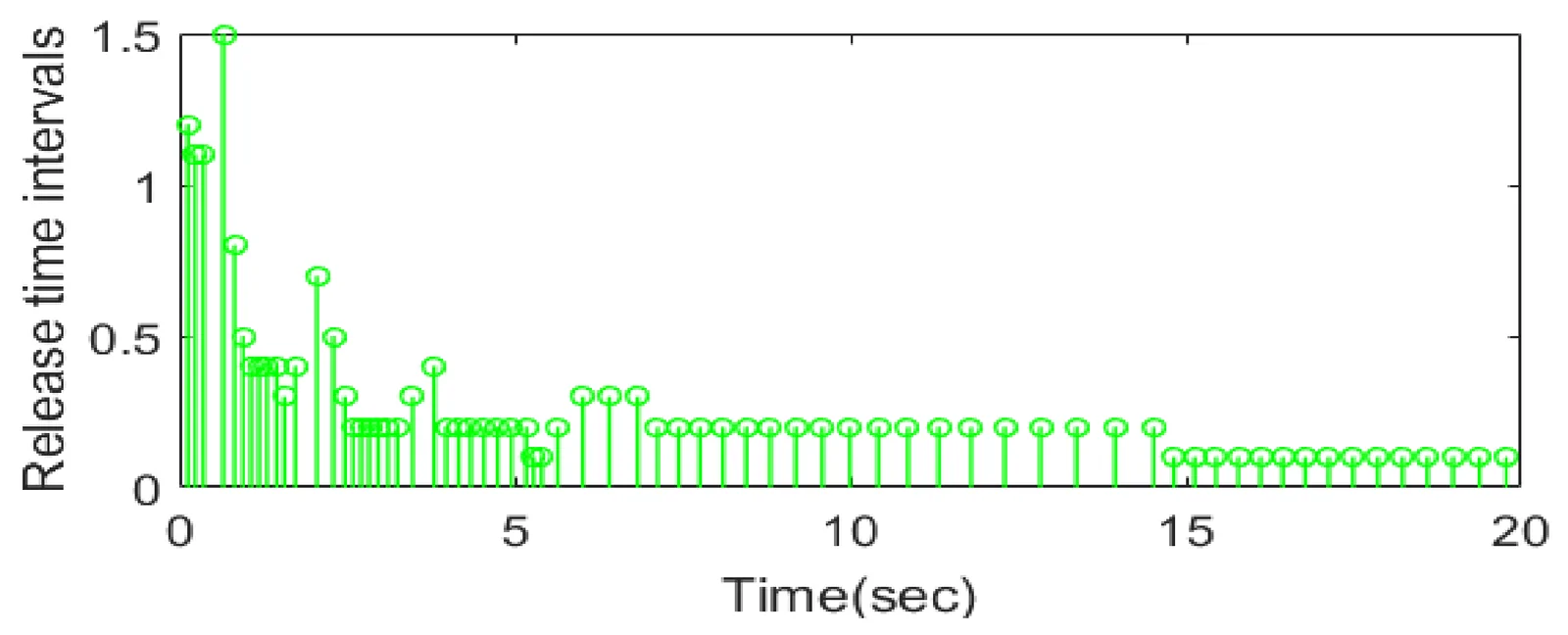
The release time intervals of the actuator channel.

**Figure 12:**
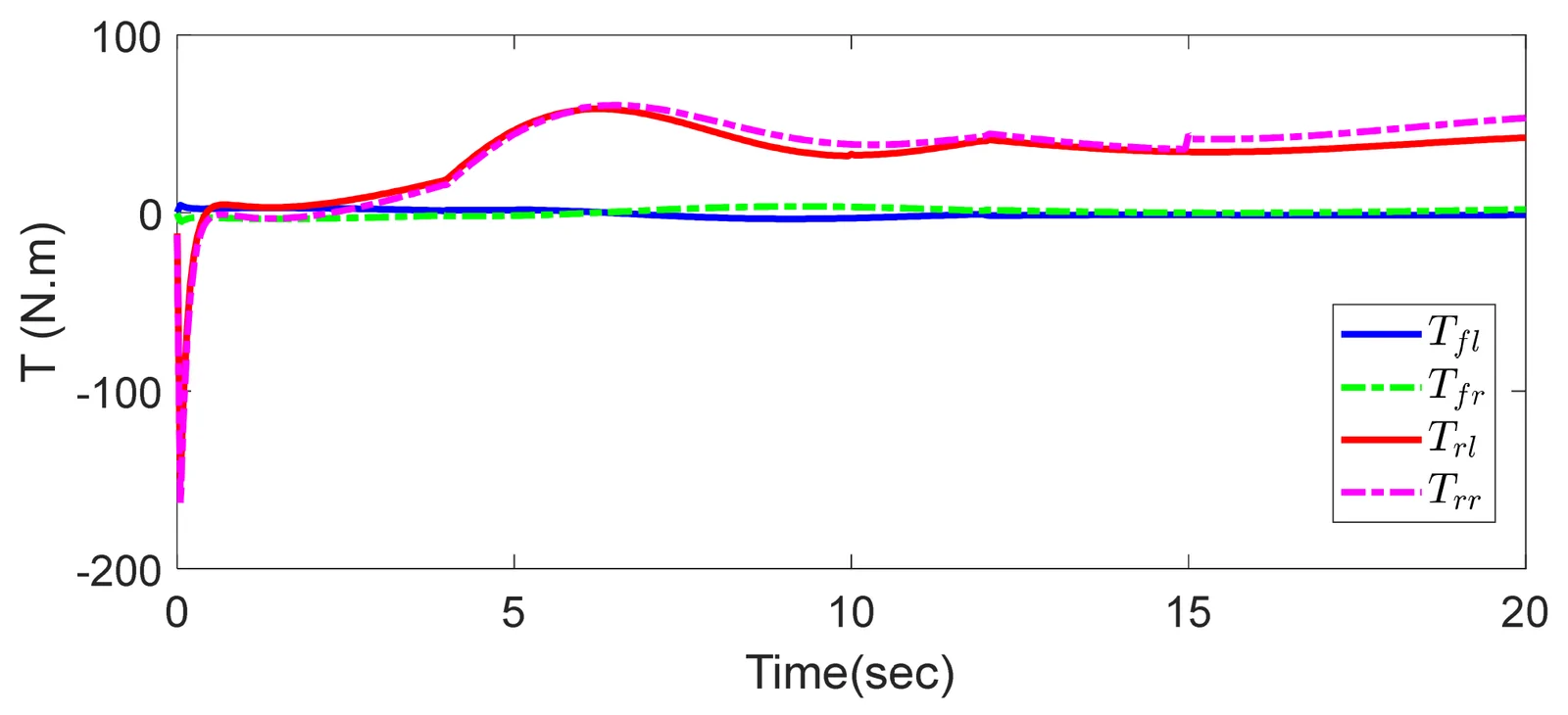
Motor output torque.

**Figure 13:**
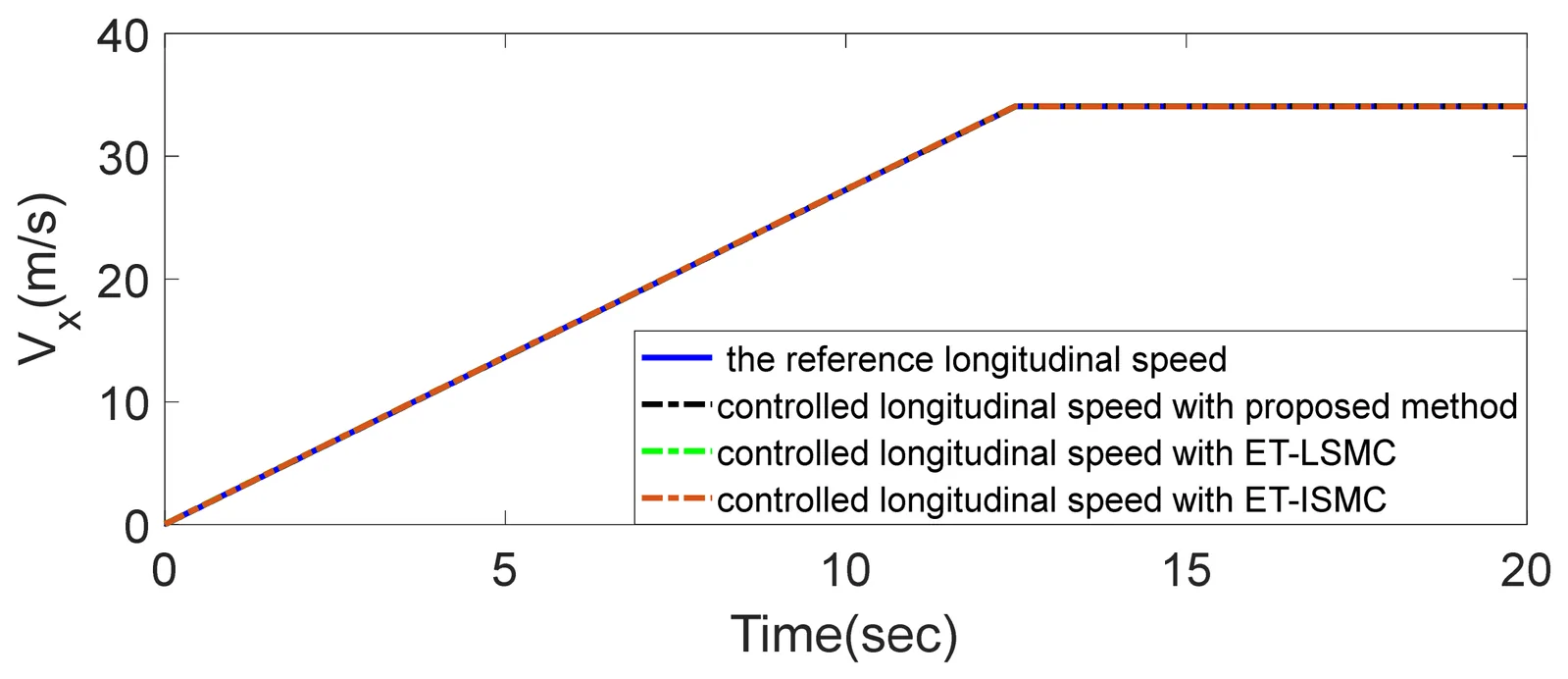
Longitudinal speed tracking results.

**Figure 14:**
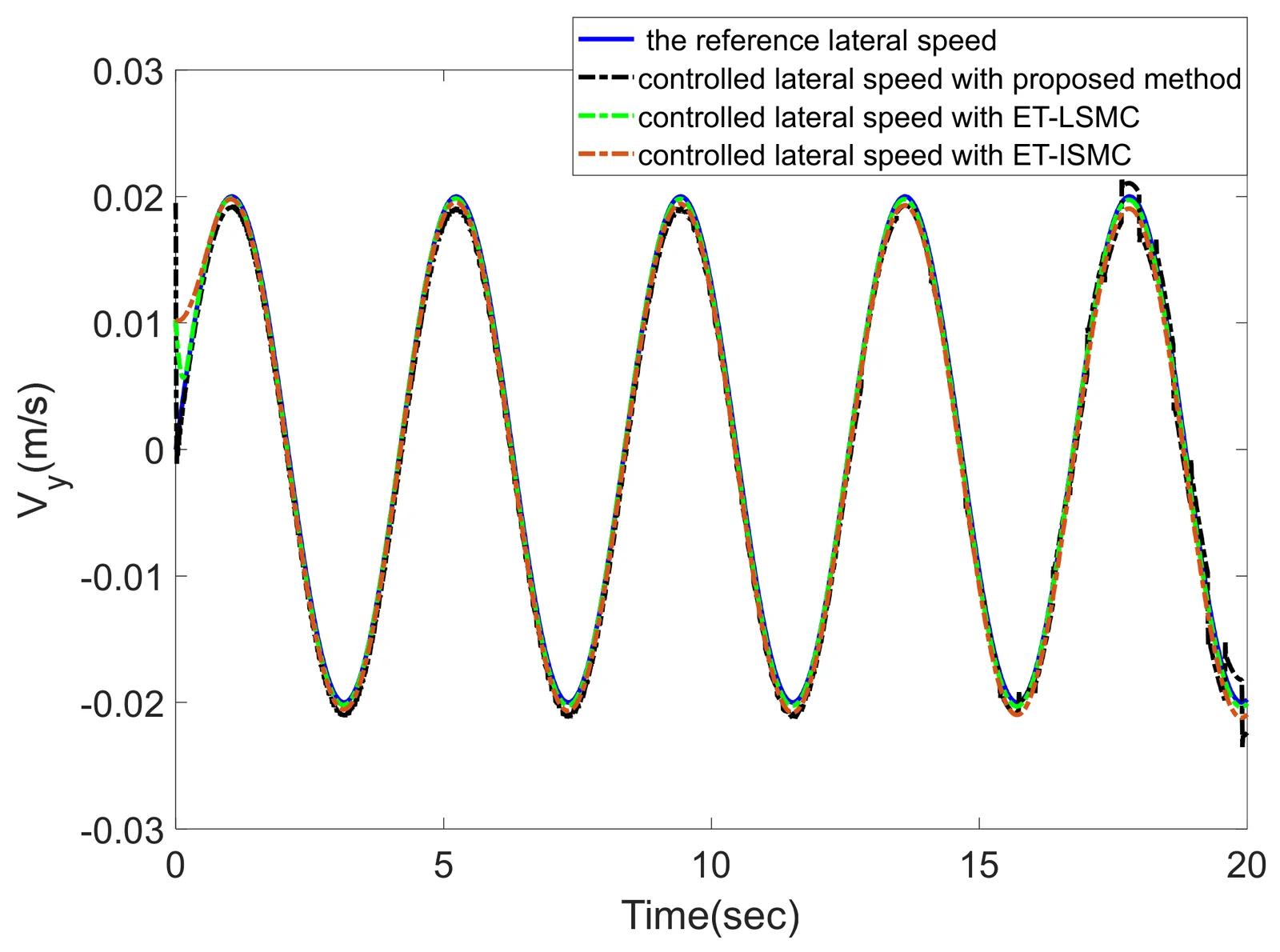
Lateral speed tracking results.

**Figure 15:**
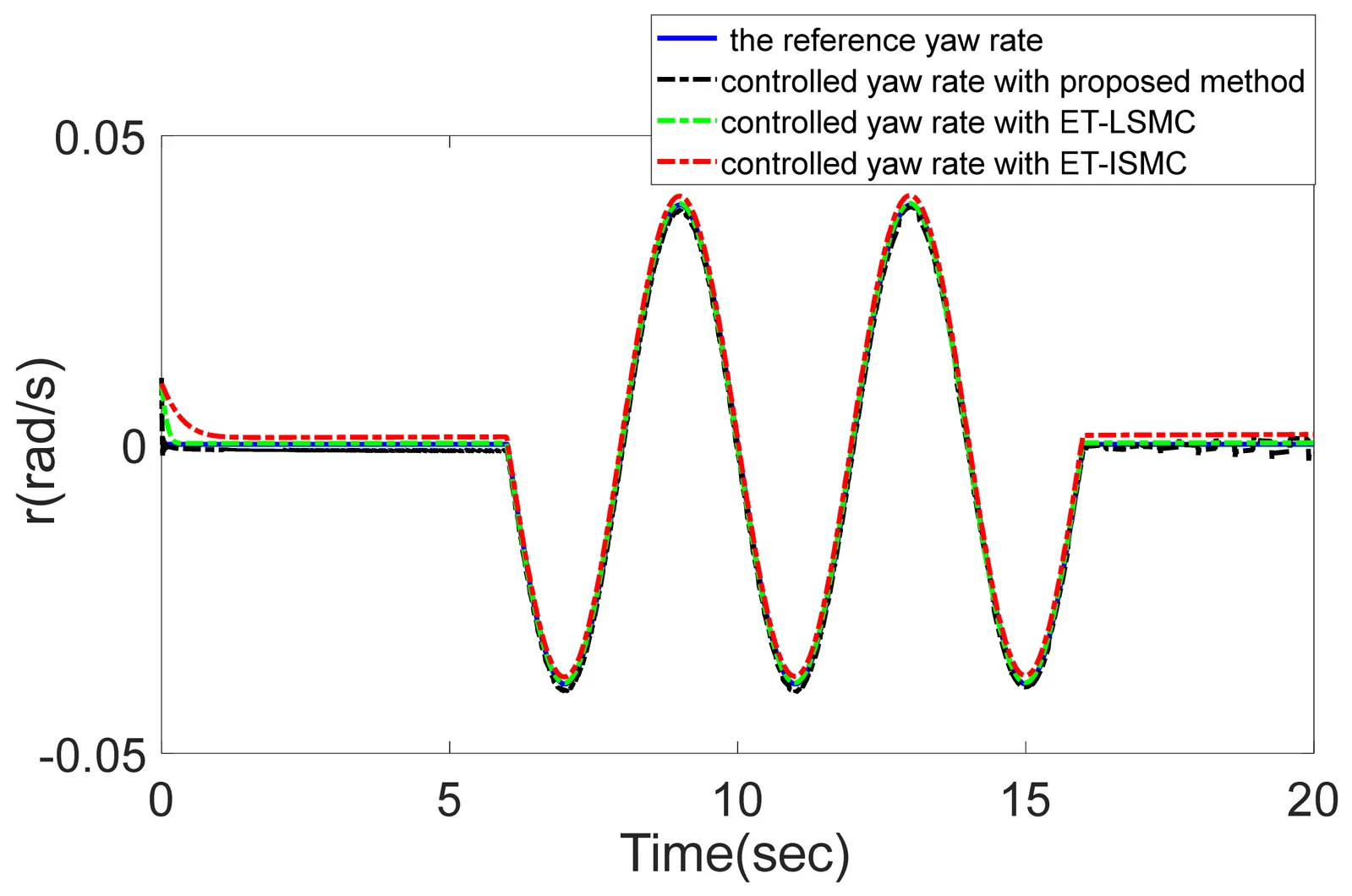
Yaw rate tracking results.

**Figure 16:**
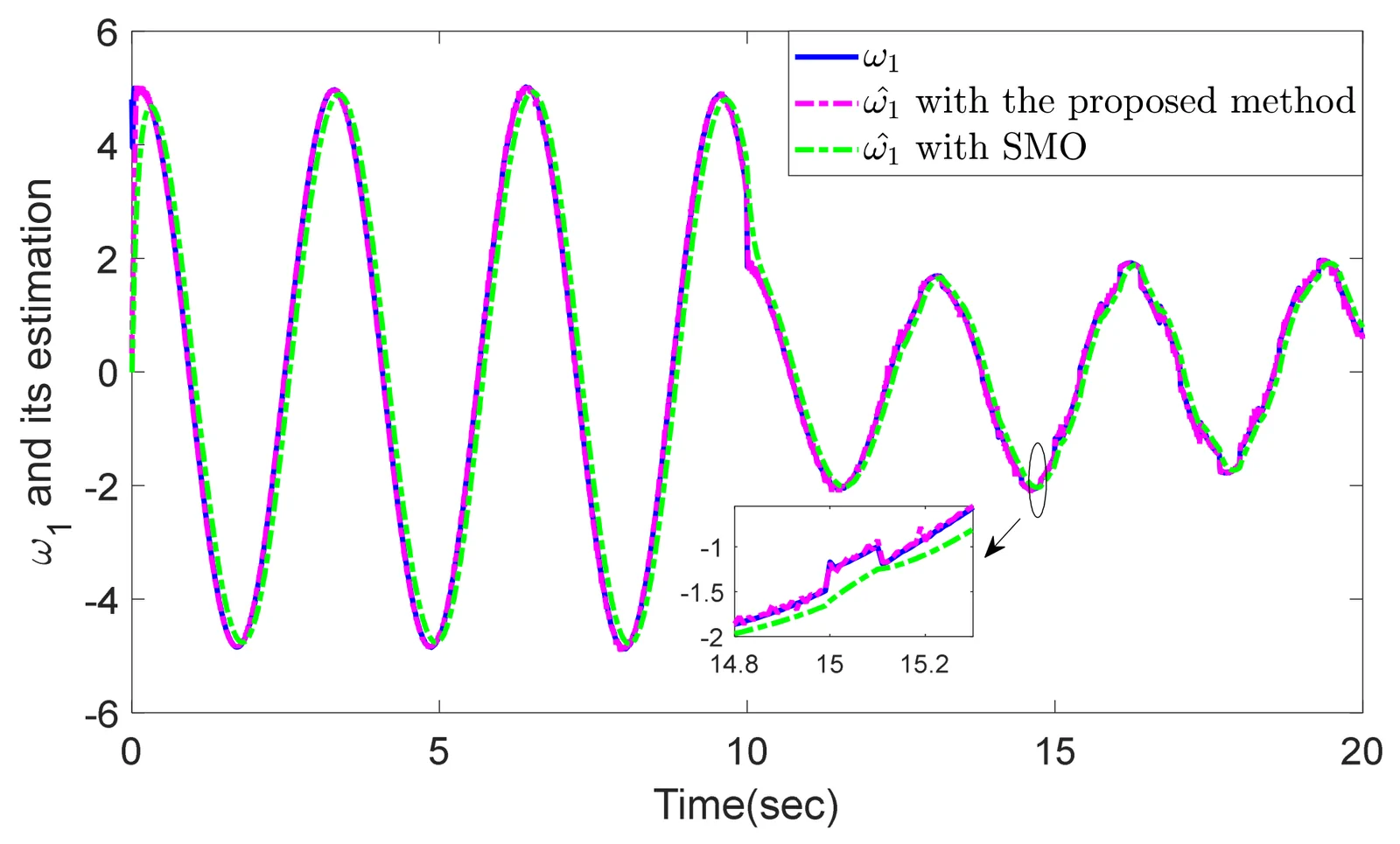
$w_{1}$ and its estimation.

**Figure 17:**
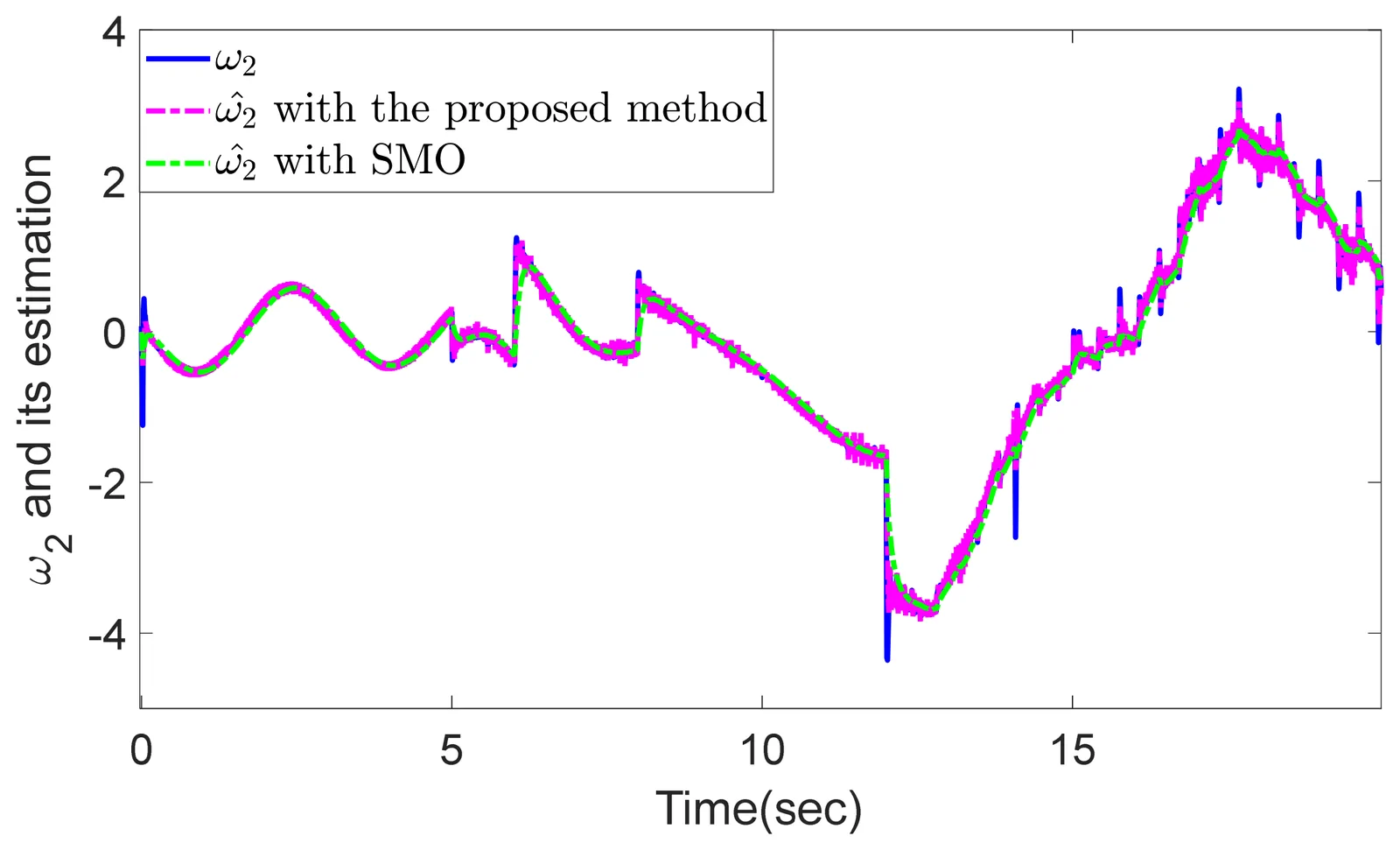
$w_{2}$ and its estimation.

**Figure 18:**
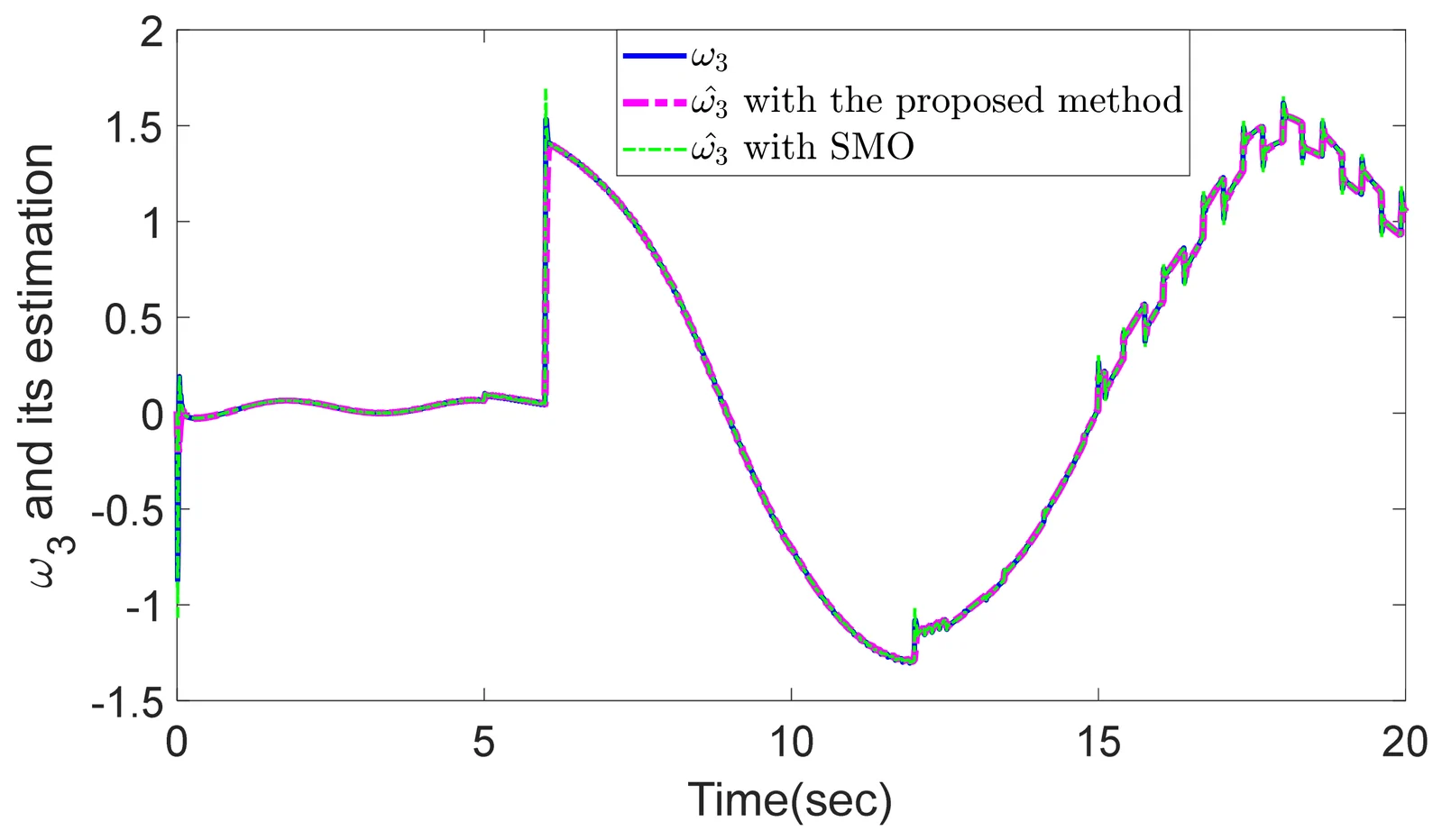
$w_{3}$ and its estimation.

**Figure 19:**
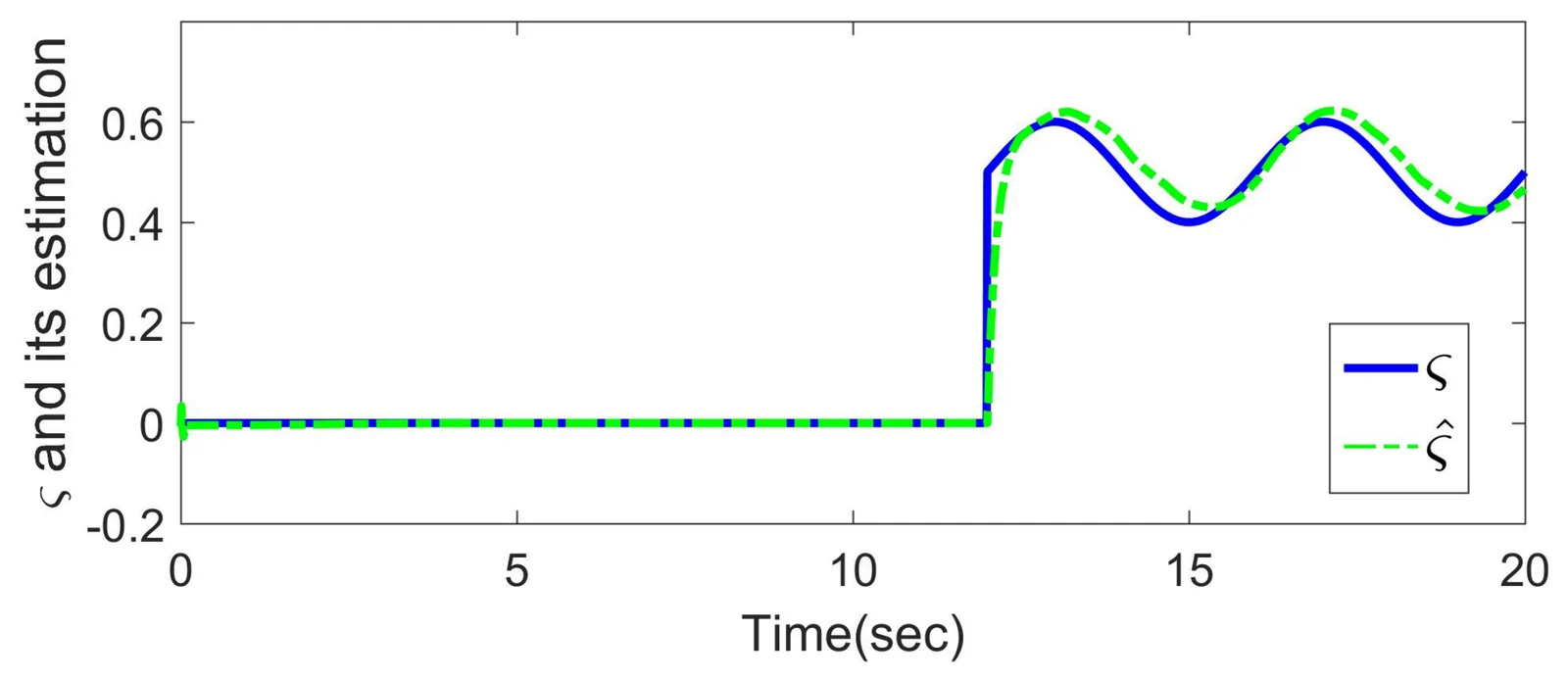
ς and its estimation.

**Figure 20:**
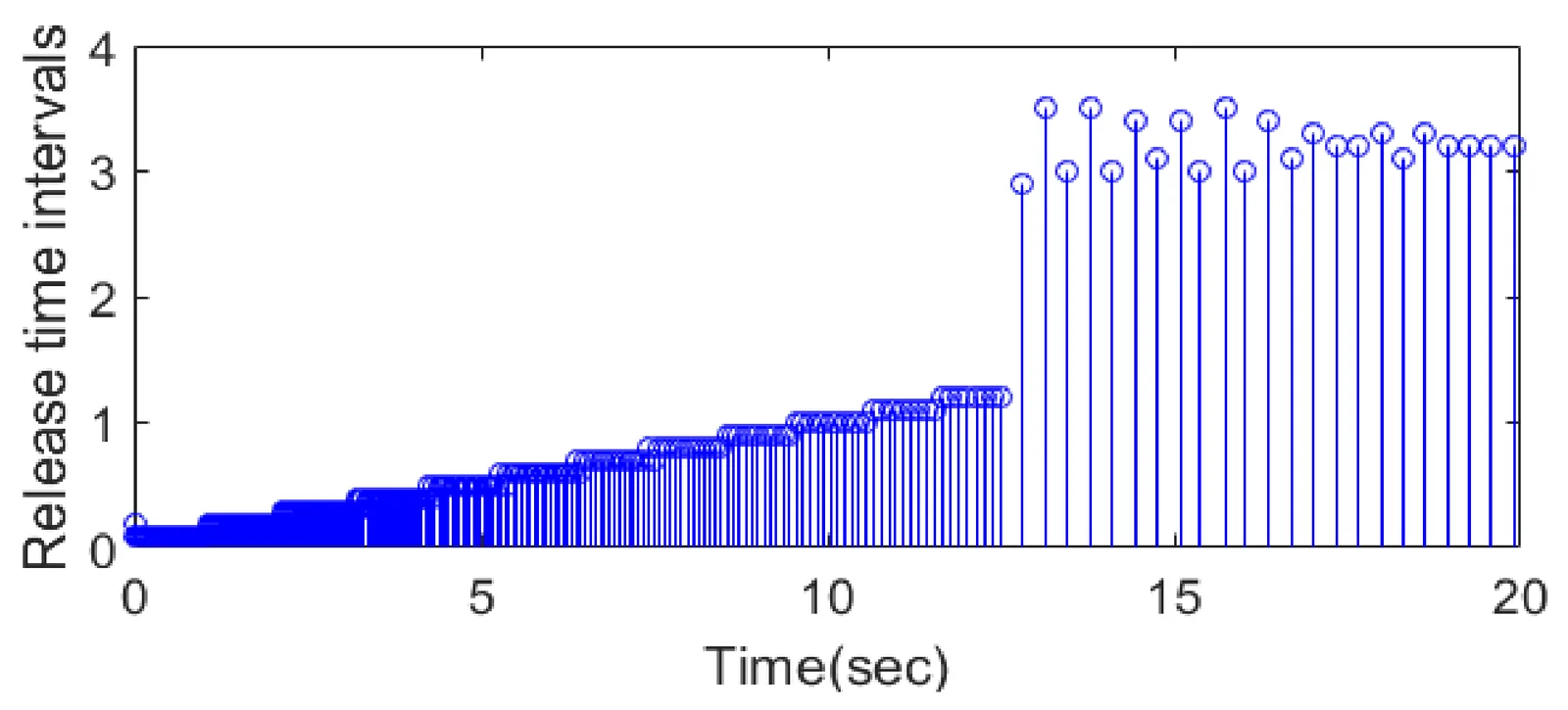
The release time intervals of y.

**Figure 21:**
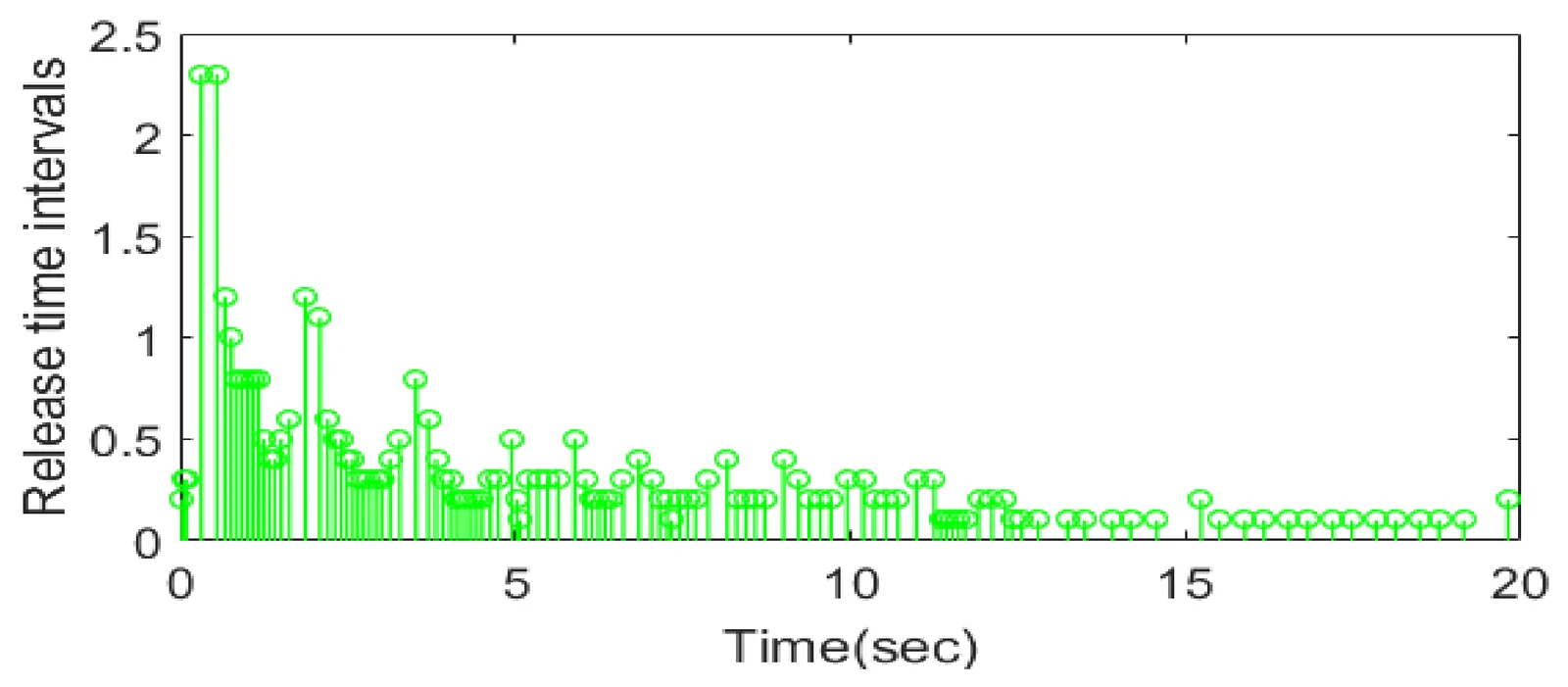
The release time intervals of u.

**Figure 22:**
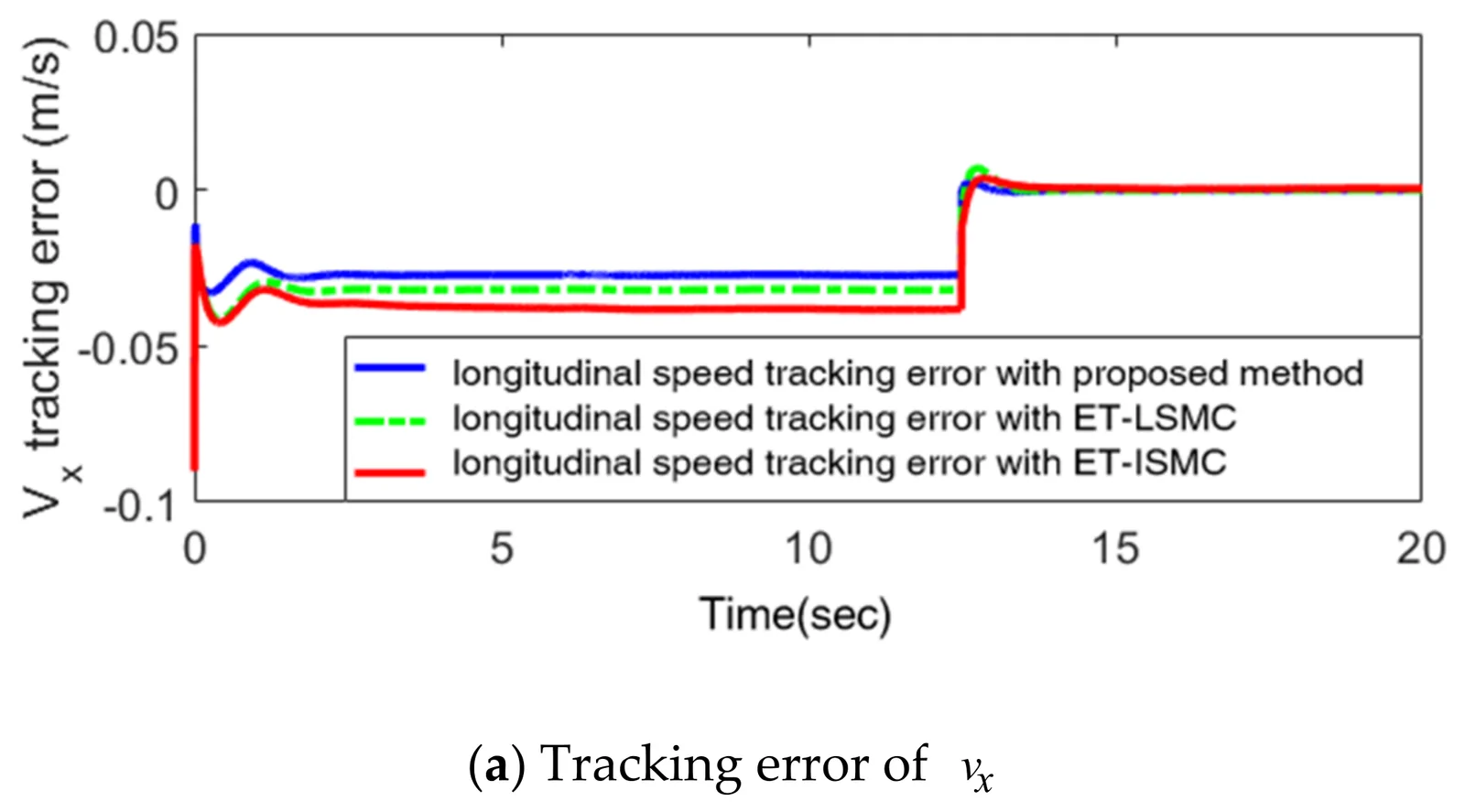

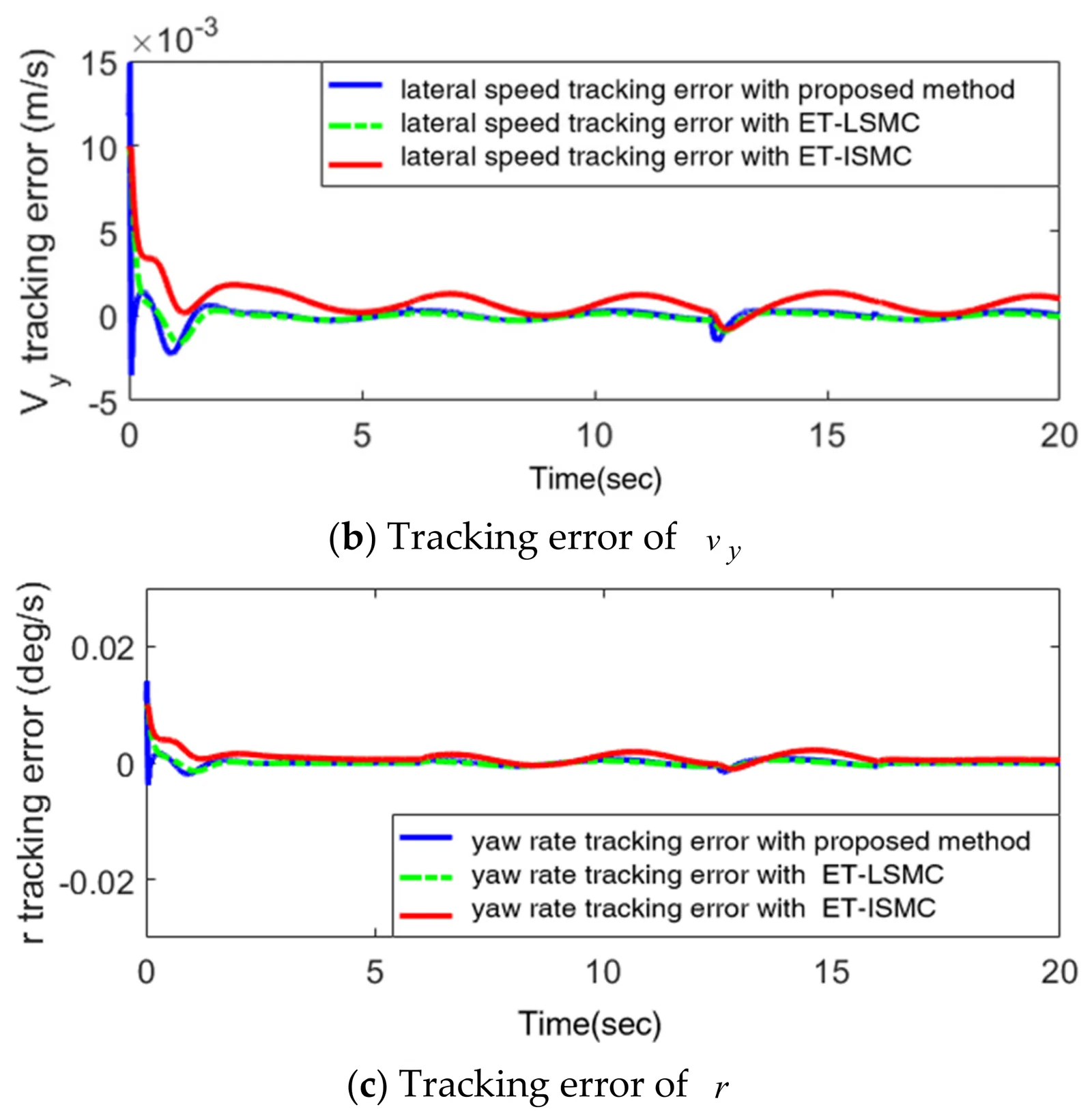
Tracking errors with different methods.

**Figure 23:**
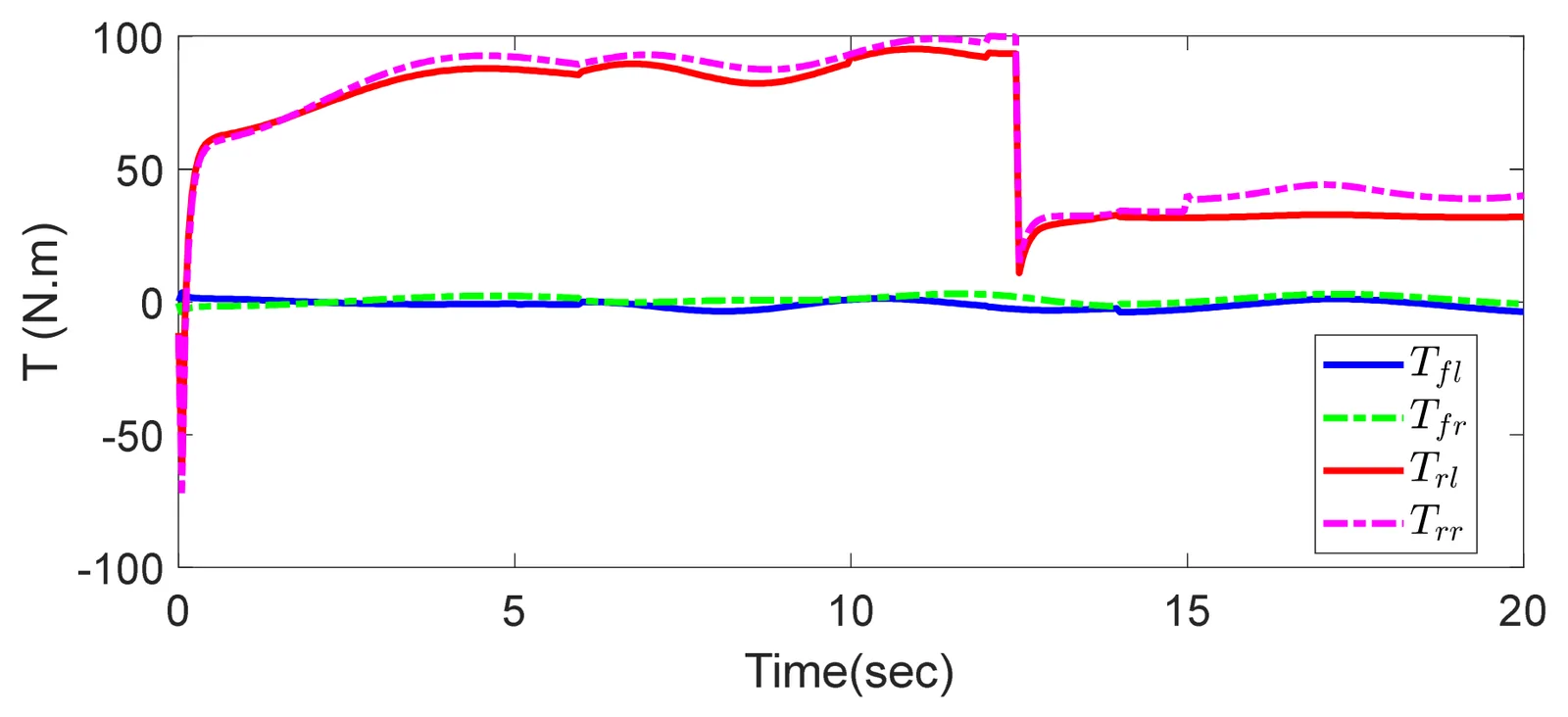
The output motor torque.

**Table 1:** Comparison results of different methods.

State	$e_{1}$		$e_{2}$		$e_{3}$	
Criterion	RMSE	Maximum Tracking Error	RMSE	Maximum Tracking Error	RMSE	Maximum Tracking Error
Proposed method	0.0077	0.004	0.0008	0.001	0.0006	0.0001
TSMFTC	0.0128	0.011	0.0024	0.003	0.0014	0.01
FTSMFTC	0.0106	0. 014	0.0013	0.01	0.0005	0.02

**Table 2:** Comparison results of different methods.

State	$e_{1}$		$e_{2}$		$e_{3}$	
Criterion	RMSE	Maximum Tracking Error	RMSE	Maximum Tracking Error	RMSE	Maximum Tracking Error
Proposed method	0.0219	0.005	0.0007	0.001	0.0008	0.002
In [29]	0.0255	0.02	0.0030	0.002	0.0030	0.005
In [22]	0.0297	0.01	0.0.0013	0.005	0.0014	0.01

## Data Availability

The datasets presented in this article are not readily available because the data are part of an ongoing study.

## Nomenclature List

$V_{x}$	Longitudinal speed
$V_{y}$	lateral speed
r	yaw rate
${F_{i}}^{x}$	tire forces of the longitudinal orientation
$F_{i}^{y}$	tire forces of lateral orientation
$l_{f}$	the distance between the front wheel and the center of the vehicle
$l_{r}$	the distance between the rear wheel and the center of the vehicle
$l_{s}$	the one-half distance between the front two wheels
m	the vehicle mass
φ	weighting matrix of the event detector
L	observer gain
H	observer gain
$H_{1}$	observer gain
P	positive symmetry matrix
Q	positive symmetry matrix
$\varphi_{a}$	positive scalar
$\varphi_{b}$	positive scalar
